# Acid-Modified and Surfactant-Functionalized Bentonite and Clinoptilolite for Naproxen Adsorption from Water

**DOI:** 10.3390/molecules31203636

**Published:** 2026-10-12

**Authors:** Gloria Lourdes Dimas Rivera, Mario Osvaldo Caballero Leal, Teresa de Jesús Montalvo Herrera, Ladislao Sandoval Rangel, David Alejandro De Haro Del Río

**Affiliations:** 1 Facultad de Ciencias Químicas, Universidad Autónoma de Nuevo León, Av. Universidad S/N, San Nicolás de los Garza 66450, NL, Mexico; 2 Tecnologico de Monterrey, Instituto de Materiales Avanzados para la Manufactura Sostenible, Av. Eugenio Garza Sada 2501, Monterrey 64849, NL, Mexico; 3 Tecnologico de Monterrey, Escuela de Ingeniería y Ciencias, Ave. Eugenio Garza Sada 2501 Sur, Monterrey 64700, NL, Mexico; 4 Ingeniería en Diseño Industrial, Universidad Politécnica de Apodaca, Av. Politécnica No. 2331, Col. El Barretal, Apodaca 66600, NL, Mexico

**Keywords:** naproxen adsorption, natural zeolite, bentonite, HDTMA surfactant modification, acid activation, pharmaceutical removal

## Abstract

Pharmaceutical contamination of aquatic environments, particularly by nonsteroidal anti-inflammatory drugs such as naproxen, remains a persistent challenge for conventional water treatment. This study evaluated the naproxen adsorption performance of bentonite and clinoptilolite in their natural, acid-activated, and hexadecyltrimethylammonium (HDTMA)-modified forms. Materials were characterized by Fourier-transform infrared spectroscopy (FTIR), X-ray diffraction (XRD), and nitrogen physisorption, confirming that acid activation increased surface area and porosity while HDTMA introduced an organophilic character without disrupting the crystalline framework. Adsorption experiments identified pH 6 as optimal for all HDTMA-modified materials. Sequentially modified clinoptilolite (C-AS) achieved the highest capacity, reaching 14.8, 16.5, and 19.9 mg g^−1^ at 25, 30, and 35 °C, respectively, outperforming all bentonite-based materials, whose interlayer pore blockage limited surfactant benefits. Kinetic data were best described by the pseudo-second-order model for most materials, while the Elovich and Avrami fractional-order models provided superior fits overall, with sub-unitary Avrami exponents indicating a heterogeneous, multi-pathway adsorption mechanism; Weber–Morris analysis further confirmed that intraparticle diffusion was not the sole rate-limiting step. Equilibrium data showed mixed Langmuir–Freundlich behavior that was resolved using the Sips model, and thermodynamic parameters derived from a three-temperature (25–35 °C) study confirmed spontaneous, endothermic adsorption governed by physisorption. These results demonstrate that the pairing of acid activation with surfactant functionalization is markedly more effective for zeolitic frameworks than for layered clays, offering a low-cost strategy for pharmaceutical removal from water.

## 1. Introduction

Over the past two decades, the presence of pharmaceutical residues in aquatic environments has emerged as one of the most persistent challenges in water-quality management. Unlike conventional pollutants, these compounds are continuously introduced through human and veterinary use, and their chemical stability often allows them to bypass the treatment processes originally designed for municipal wastewater [1]. Nonsteroidal anti-inflammatory drugs (NSAIDs) such as ibuprofen, ketoprofen, diclofenac, and naproxen have been detected in wastewater at concentrations reaching several thousand nanograms per liter [2]. Naproxen is among the most consistently reported pharmaceuticals in surface water and wastewater treatment plants worldwide, with concentrations in surface waters reaching up to 32 μg L^−1^ in some regions [3]. Its limited biodegradability and incomplete removal during conventional treatment mean that, once released, naproxen tends to persist and accumulate in the aquatic environment [4], raising concern over chronic ecotoxicological effects and the long-term implications of low-level human exposure through drinking water [5].

Several technologies have been explored to address this problem, each with its own trade-offs. Advanced oxidation processes (AOPs) demonstrate strong efficacy in pharmaceutical degradation but are limited by high-energy requirements and the formation of potentially toxic by-products [6,7]. Adsorption, by contrast, offers advantages in efficiency, operational simplicity, cost-effectiveness, and scalability, with activated carbon serving as the benchmark adsorbent [8]. Nevertheless, the costs of the production process and regeneration have promoted the exploration of low-cost alternatives, which combine chemical stability, availability, and suitable adsorption properties [9]. Among these materials, natural clays and zeolites have attracted particular attention owing to their abundance, low cost, and favorable structural properties. Bentonite, a clay dominated by montmorillonite, possesses a layered aluminosilicate structure and an enhanced specific surface area that make it a versatile sorbent for a wide range of pollutants [10]. Clinoptilolite, one of the most common natural zeolites, offers a three-dimensional framework of interconnected channels and cavities together with a substantial cation-exchange capacity [11]. Both materials have been extensively studied for the removal of heavy metals and other organic contaminants. Despite these favorable properties, their inherently hydrophilic surfaces limit their affinity for weakly polar or neutral organic molecules such as naproxen, restricting the adsorption capacities achievable using natural materials [12,13].

To overcome this limitation, two complementary modification strategies have been widely adopted. Acid activation replaces interlayer cations with protons and partially leaches structural Al, Fe, and Mg, which increases specific surface area and porosity while preserving the overall crystalline framework [14,15]. Reported increases in surface area following acid treatment of bentonite range from roughly two- to sixfold, depending on acid concentration, temperature, and treatment time. Building on this activated surface, modification with cationic surfactants such as hexadecyltrimethylammonium (HDTMA) introduces an organophilic character through cation exchange, forming a hydrophobic surface layer capable of partitioning organic compounds via van der Waals interactions in addition to electrostatic mechanisms [16]. The individual effects of these modifications have been documented in related contexts: HDTMA-modified bentonite has shown substantially enhanced adsorption of per- and polyfluoroalkyl substances compared with the untreated material, while surfactant-modified bentonite has improved the removal of ibuprofen and diclofenac sodium, reaching adsorption capacities above 100 mg g^−1^ under optimized surfactant loading [17]. HDTMA modification of clinoptilolite has also been shown to preserve the zeolitic crystalline framework while forming an organic surface layer that modestly improves the adsorption of small pharmaceutical molecules relative to the natural material. However, most of these studies examine bentonite and clinoptilolite separately, address structurally different pharmaceuticals, or apply surfactant modification without a preceding acid-activation step. A systematic, side-by-side comparison of these two natural aluminosilicates, subjected to the same acid-activation and HDTMA-modification sequence and evaluated specifically for naproxen removal, has, to the best of our knowledge, not yet been reported.

The aim of this study was to evaluate and compare the naproxen adsorption performance of bentonite and clinoptilolite, both in their natural form and after sequential acid-activation and HDTMA modification. Acid-treated samples were included not as passive control but as an intermediate treatment stage, allowing the individual contribution of acid activation to be distinguished from that of subsequent HDTMA functionalization in the sequentially modified materials. The natural, acid-treated, and surfactant-modified materials were characterized by X-ray diffraction (XRD), Fourier-transform infrared spectroscopy (FTIR), and nitrogen physisorption to assess their mineralogical, chemical, and textural properties. Adsorption experiments were carried out to evaluate the effect of solution pH, contact time, and initial naproxen concentration. The resulting kinetic data were fitted to the pseudo-first-order (PFO), pseudo-second-order (PSO), Avrami, Elovich, and Weber–Morris intraparticle diffusion models, while equilibrium data obtained at different temperatures were analyzed using the Langmuir, Freundlich, and Sips isotherm models to elucidate the mechanisms governing naproxen removal on each material.

## 2. Results and Discussion

### 2.1. Characterization

Figure 1 shows the X-ray diffraction (XRD) patterns of natural bentonite (B), natural clinoptilolite (C), and their acid-treated and HDTMA-modified counterparts. Figure 1a shows the diffraction patterns of the bentonite-based materials. The diffraction pattern of the natural bentonite (B) exhibits the characteristic crystalline structure of montmorillonite, as confirmed by the reflections at 2θ ≈ 6° (001), 20° (100), 35° (110), and 55° (210), together with minor peaks from illite (≈22°) and quartz (≈28, 46, and 52°) [18]. In the acid-treated and surfactant-modified samples (B-A and B-AS), these reflections remain at essentially unchanged positions, with only a slight decrease in intensity and moderate broadening of the (001) peak near 6°. The characteristic montmorillonite reflections remained detectable across all samples, indicating that neither HCl activation nor HDTMA modification disrupted the clay’s overall layered framework. The only notable change was a slight shift of the (001) reflection toward lower 2θ values in the HDTMA-modified samples (B-S and B-AS). This shift is consistent with the intercalation of HDTMA cations into the interlayer space, which expands the basal spacing without compromising the layer stacking [19].

For the clinoptilolite-based materials (Figure 1b), the natural sample C matches JCPDS cards No. 00-025-1349 and 01-079-1460, with main reflections at 2θ ≈ 10° (020), 13° (−201), 17° (111), 23° (−421), and 26° (−222) [20]. An additional diffraction peak at 2θ = 29.26° is attributed to a minor crystalline impurity that disappears after acid treatment (C-A). In addition, weak quartz-related peaks near 36° and 40° were also detected. HDTMA modification (C-AS) produces a slight decrease in the intensity of the (020) reflection near 10°, consistent with surfactant adsorption rather than structural degradation. The preservation of the characteristic zeolitic reflections after both acid and surfactant treatments confirms that these modifications maintain the crystalline framework of the material.

Figure 2 presents the FTIR spectra of the natural, acid-treated, and HDTMA-modified bentonite and clinoptilolite samples. The bentonite spectra in Figure 2a display the typical fingerprint of a layered aluminosilicate: a sharp band at 3614 cm^−1^ assigned to structural Al–OH stretching in the octahedral sheet, a band at 1628 cm^−1^ from adsorbed water bending, and the dominant Si–O stretching of the tetrahedral sheet at 1024 cm^−1^. Additional bands at 945, 796, 623, and 515 cm^−1^, corresponding to Al–O, Si–O, and Si–O–Al vibrations, complete the montmorillonite signature [21]. After HDTMA modification, samples B-S and B-AS show two weak but distinct bands at 2855 and 2927 cm^−1^, arising from symmetric and asymmetric C–H stretching of the surfactant alkyl chains, more clearly resolved in the magnified spectrum (Figure 2b) [22].

The clinoptilolite samples (C, CA, C-S, C-AS) show in Figure 2c a weak water-bending band near 1640 cm^−1^ and a calcite-related band at 1450 cm^−1^, reflecting a natural impurity that is characteristic of untreated clinoptilolite [23]. The zeolitic framework itself is evident from bands at 1040 cm^−1^ (Si–O–Si asymmetric stretching), 790 and 450 cm^−1^ (Si–O and O–Si–O vibrations), and 597 cm^−1^ (Al–O–Si torsion). As with bentonite, HDTMA loading introduces C–H stretching bands at approximately 2850 and 2930 cm^−1^ in C-S and C-AS (Figure 2d), confirming surfactant anchoring on the zeolite surface [24]. These results indicate that modification with HDTMA preserves the aluminosilicate framework of bentonite and clinoptilolite, while the appearance of characteristic C–H stretching bands confirms successful surfactant incorporation onto both materials.

The textural properties of clinoptilolite and bentonite, before and after HCl and HDTMA treatment, are summarized in Table 1. The N_2_ adsorption–desorption isotherms in Figure 3 reflect the structural framework of each mineral: clinoptilolite showed a pronounced uptake at low relative pressures (P/P_0_ < 0.05), indicative of micropore filling, together with an H4-type hysteresis loop typical of narrow slit-shaped micropores, whereas bentonite displayed an H3-type loop associated with aggregates of plate-like particles and a greater mesoporous contribution [1,2].

Acid treatment enhanced the textural properties of both minerals, though far more markedly for clinoptilolite, whose BET surface area, micropore area, and total pore volume increased by approximately 258%, 1090%, and 90%, respectively, relative to the natural material. Bentonite showed comparatively modest gains (40%, 42%, and 32% for the same parameters). This contrast reflects the different mechanisms by which HCl acts on each structure: in clinoptilolite, the pronounced increase in microporosity is consistent with partial dealumination of the framework and the clearing of extra-framework species that block the channels; direct compositional confirmation (e.g., Si/Al ratio via XRF or EDS) was not obtained in this study and is noted as a limitation below, whereas in bentonite it is suggested that it mainly removes impurities and leaches octahedral cations without substantially altering the layered structure [3,5].

HDTMA modification produced contrasting effects depending on the mineral. In clinoptilolite (C-S), surfactant loading increased the BET surface area by ~20% and total pore volume by ~33%, yet nearly eliminated the micropore area, suggesting that HDTMA molecules preferentially deposit at the channel entrances and obstruct N_2_ access [4]. When applied after acid treatment (C-AS), both surface area and microporosity decreased further, indicating that the surfactant occupies part of the porosity previously opened by dealumination. Bentonite behaved oppositely: HDTMA alone (B-S) produced the largest textural gains of all samples (204% in BET area, 236% in pore volume), consistent with surfactant-induced interlayer expansion, while sequential acid–HDTMA treatment (B-AS) roughly halved this gain, suggesting partial pore occupation by the surfactant after the layered structure had already been altered by acid activation.

Pore-size distributions can be seen in Figure 4 which further clarifies the textural changes from acid-activation and HDTMA modification. In bentonite, pores were concentrated in the mesoporous range (1–15 nm); natural bentonite (B) showed a broad distribution centered at 3–7 nm, consistent with interparticle and interlayer spacing [1]. Acid activation (B-A) intensified a contribution near 2 nm, at the micro/mesopore boundary, reflecting cation leaching and improved pore accessibility [3], which decreased after HDTMA modification (B-S, B-AS). This result is consistent with partial surfactant occupation of interlayer space rather than pore destruction [4]. For clinoptilolite, the natural sample (C) showed hierarchical porosity, with microporous maxima at 0.5–0.7 and 1.5–1.8 nm alongside mesoporosity at 6–10 nm, reflecting the zeolitic channel system [6]. Acid treatment (C-A) intensified the ~0.55–0.60 nm contribution and extended mesoporosity to ~15 nm [7], while HDTMA modification (C-S, C-AS) reduced sub-1-nm porosity in favor of a ~1.8 nm contribution and extended mesoporosity up to ~26 nm. These results confirm that acid-activation and HDTMA functionalization differentially reshape micro- and mesoporous accessibility in both minerals, consistent with the BET and microporosity results previously described.

### 2.2. Adsorption Results

#### 2.2.1. pH

Naproxen [(S)-6-methoxy-α-methyl-2-naphthaleneacetic acid] is a propionic-acid-derivative NSAID bearing a carboxylic acid group attached to a methoxy-substituted naphthalene ring. As a weak monoprotic acid with a pKa of approximately 4.2 [25], its speciation in aqueous solution is strongly pH-dependent: below pH ≈ 3, naproxen remains predominantly in its neutral, protonated form (–COOH), favoring hydrogen-bonding interactions with silanol and Al–OH surface groups; above pH ≈ 5, it exists almost entirely as the anionic carboxylate species (–COO^−^), favoring electrostatic interactions with cationic surface sites [3]. This pH-dependent speciation underlies the adsorption behavior discussed below.

Figure 5 illustrates the adsorption behavior of clinoptilolite- and bentonite-based materials as a function of pH. For clinoptilolite-derived materials, C-AS reaches its highest adsorption capacity at pH 6 (16.3 mg g^−1^), where naproxen is fully deprotonated. This promotes strong electrostatic attraction with positively charged quaternary ammonium groups (HDTMA), further enhanced by hydrophobic partitioning into admicellar and hemimicellar domains [26]. In contrast, C-A exhibits its maximum adsorption at pH 4 (14.3 mg g^−1^), which decreases at higher pH. At this condition, a significant fraction of naproxen remains in its neutral form, favoring hydrogen bonding with silanol (Si–OH) groups generated during acid treatment. Although C-A showed its highest adsorption capacity at pH 4, a similar value was obtained at pH 6. Therefore, pH 6 was selected for subsequent kinetic and equilibrium studies to enable direct comparison among all materials.

Similarly, bentonite-based materials (B-S and B-AS) display maximum adsorption at pH 6 (6.6 and 6.5 mg g^−1^, respectively), driven by Coulombic attraction between naproxen anions and intercalated HDTMA head groups, along with hydrophobic interactions involving the aromatic moiety of the pharmaceutical [27]. However, a different behavior occurs at alkaline pH: while B-S retains moderate adsorption at pH 8 (4.3 mg g^−1^), B-AS shows a sharp decline to near-zero capacity (0.05 mg g^−1^). This behavior suggests that acid pretreatment in B-AS induces structural cation leaching and destabilization of edge-site charges, which under alkaline conditions enhances OH^−^ competition and disrupts surfactant layer organization, ultimately hindering adsorption [28]. Overall, under more alkaline conditions (pH 8–10), all materials exhibit reduced adsorption capacities possibly due to dominant electrostatic repulsion between negatively charged surfaces and naproxen anions, combined with increased competition from OH^−^ ions for active sites [29].

#### 2.2.2. Kinetics

Figure 6 shows the adsorption kinetics of naproxen onto bentonite and its modified counterparts. B-A showed the greatest adsorption performance, attaining an adsorption capacity of nearly 7.5 mg g^−1^ and reaching equilibrium after approximately 100–150 min. Notably, this material exhibited a pronounced affinity for naproxen from the earliest contact periods, indicating a strong initial affinity toward naproxen, followed by a slower diffusion-controlled uptake within the expanded interlayer structure. Conversely, B-S and B-AS displayed significantly lower adsorption capacities (2.5–4.4 mg g^−1^) and achieved equilibrium within the first 50 min. The kinetic profiles of these materials followed an exponential ascending trend, characterized by a rapid initial uptake phase and a subsequent stabilization as the adsorption sites became progressively occupied. These differences can be attributed to the physical blockage of interlayer spaces by intercalated HDTMA molecules, which restrict mass transfer [30]. Natural bentonite (B) did not exhibit any measurable naproxen uptake, highlighting the critical role of surface modification in enhancing adsorption performance.

For the clinoptilolite series, in Figure 6b, C, C-A, and C-S reached equilibrium within 50 min, indicating rapid occupation of accessible adsorption sites. In contrast, C-AS exhibited a different kinetic profile, with a continuous increase in adsorption capacity, surpassing 16.8 mg g^−1^ after 200 min before reaching equilibrium. This enhanced performance can be attributed to the synergistic effect of the acid–surfactant treatment. Acid activation removes exchangeable cations and extra-framework species, increasing pore accessibility and exposing additional adsorption sites, while facilitating the incorporation of HDTMA within the zeolitic structure and promoting prolonged intraparticle diffusion [31]. In contrast to bentonite, where surfactant loading may occupy interlayer spaces and reduce site accessibility, the rigid clinoptilolite framework benefits from acid-induced channel opening, enabling a synergistic interaction between pore development and surfactant functionalization [12].

The kinetic adsorption data for both bentonite and clinoptilolite materials were fitted to four kinetic models: pseudo-first-order (PFO), pseudo-second-order (PSO), Elovich, and Avrami fractional order. The resulting parameters, together with the R^2^ and chi-square (χ^2^) goodness-of-fit statistics, are summarized in Table 2 and Table 3. For the bentonite series (Figure 7), the PSO model provided the best fit among PFO and PSO for all three modified materials, with correlation coefficients R^2^ of 0.9422, 0.9528, and 0.9091 for B-A, B-S, and B-AS, respectively. Notably, acid-activated B-A exhibited the lowest PSO rate constant (K_2_ = 0.0001 g mg^−1^ min^−1^) but the highest capacity, reflecting a slow, swelling-driven intraparticle diffusion into the deep interlayer galleries [32]. Conversely, the surfactant-modified B-S and B-AS displayed higher K_2_ values (0.0022 and 0.0031 g mg^−1^ min^−1^), indicating rapid saturation limited to the external organophilic surface. This confirms that while HDTMA intercalation facilitates fast initial uptake, it blocks internal pores, reducing the total capacity [33].

The Elovich model, originally developed to describe chemisorption on energetically heterogeneous solid surfaces, assumes that the activation energy for adsorption increases linearly with surface coverage, without significant desorption over the timescale considered [34]. It provided an exceptional fit across the bentonite series (R^2^ = 0.969–0.996; Table 3), supporting a mechanism in which naproxen occupies progressively less favorable sites as adsorption proceeds. This result is consistent with a surface (external, interlayer, or HDTMA-modified) that is not energetically uniform, rather than a single class of equivalent binding sites. The correspondingly low χ^2^ values (0.014–0.203) confirm that this improvement holds even at the low-q, short-time data points, where PFO’s poorest agreement with the experimental data occurs [35].

The Avrami fractional-order model, adapted from nucleation-and-growth theory in solid-state phase transformations, allows the reaction order to take non-integer values, reflecting adsorption mechanisms that may proceed through multiple, possibly overlapping pathways rather than a single elementary step [36]. It gave the best overall fit among the four models for all three bentonite materials (R^2^ = 0.972–0.999, χ^2^ = 0.003–0.184; Table 3), and the sub-unitary exponents obtained (n_AV_ = 0.13–0.53) suggest that uptake unfolds through a combination of comparatively fast external binding and a slower, structurally constrained process this is consistent with the swelling-driven diffusion into interlayer galleries already proposed from the Weber–Morris analysis.

For the clinoptilolite series, in Figure 8, the natural sample (C) best fitted the PFO model (R^2^ = 0.9574), suggesting adsorption predominantly occurring on readily accessible surface sites with limited diffusional resistance [37]. In contrast, the acid-treated (C-A) and surfactant-modified (C-S) clinoptilolites exhibited inadequate fits for both kinetic models (R^2^ < 0.56), indicating that their uptake processes cannot be described by a single rate-limiting step. The dual-modified sample (C-AS) exhibited the best fit to the PSO model (R^2^ = 0.7883), with an experimental adsorption capacity higher than those of the other clinoptilolite series. Moreover, C-AS presented the lowest PSO rate constant (K_2_ = 0.0023 g mg^−1^ min^−1^), indicating a slower but more extensive adsorption process possibly governed by intraparticle diffusion and mass-transfer limitations [38].

The Elovich model fit natural clinoptilolite (C) comparatively weakly (R^2^ = 0.947, χ^2^ = 0.316; Table 3) relative to PFO, consistent with adsorption on a more energetically uniform, readily accessible surface prior to modification. In contrast, its markedly better performance for C-A, C-S, and C-AS (R^2^ = 0.974–0.994, χ^2^ = 0.095–0.193) indicates that both acid-activation and HDTMA functionalization introduce the kind of site-to-site energetic heterogeneity the Elovich model is built to describe [34], in agreement with the textural and FTIR evidence for structurally and chemically diverse surface environments in the modified materials. Avrami exponents obtained for the clinoptilolite series further differentiate the natural and modified samples: C returned n_AV_ ≈ 1 (0.99, R^2^ = 0.985, χ^2^ = 0.094), close to the single-step behavior implied by its strong PFO fit, whereas C-A, C-S, and C-AS all returned sub-unitary values (n_AV_ = 0.19–0.27) alongside consistently high fit quality (R^2^ = 0.977–0.993, χ^2^ = 0.101–0.171; Table 3), indicating a shift toward a multi-pathway adsorption mechanism upon modification [36]. Elovich rate parameters (α, β in Table 3) spanned several orders of magnitude across the series. This is a known feature of the model, given the logarithmic sensitivity of α to curvature in the early-time data. The model’s value here lies in its superior fit statistics rather than in the literal magnitude of its fitted constants.

The intraparticle diffusion mechanism was screened using the Weber–Morris (W–M) model (Figure 9). Following Chu et al. [39], who demonstrate that the widely used form of the W–M model (Equation (8)) lacks theoretical justification in the original Weber and Morris framework, and that neither the sign/magnitude of C nor the number or slope of linear segments can be reliably attributed to specific transport mechanisms such as boundary layer thickness and sequential film/pore/equilibrium stages. The W–M plots here are used strictly as a qualitative screening tool, i.e., a single line through the origin across the full-time range would indicate that intraparticle diffusion alone governs the process, while any deviation from this behavior such as multilinearity or a non-zero intercept indicates that intraparticle diffusion (IPD) is not the sole rate-controlling step, without further decomposition of the specific mechanisms involved. All seven materials studied showed clear deviations from a single line through the origin, confirming that IPD alone does not govern naproxen uptake in any case. This is consistent with the fractional reaction orders obtained from the Avrami model which provide a more theoretically grounded basis for inferring a composite, multi-pathway adsorption process than the W–M segment structure itself.

#### 2.2.3. Adsorption Isotherms

Figure 10 shows the naproxen adsorption isotherms for clinoptilolite and bentonite and their modifications. Unmodified natural bentonite (B) exhibited negligible adsorption capacity across the tested concentration range, consistent with the null affinity already observed in the pH and kinetics sections, whereas natural clinoptilolite (C) retained approximately 3.6 mg/g (Figure 10). At near-neutral pH, naproxen is anionic. The null adsorption on B is attributed to electrostatic repulsion between the negatively charged smectite sheets and the anionic drug [40]. Conversely, C’s extra-framework cations (Ca^2+^, Mg^2+^) act as electrostatic bridges to bind the drug’s carboxylate groups [41]. Acid activation increased the capacity of both materials but with different isotherm shapes. B-A reached 7.3 mg/g with an accelerating curve, while C-A approached 8.4 mg/g with continuous growth. Textural analysis confirms that acid increased the BET area of bentonite from 39 to 55 m^2^/g, whereas clinoptilolite surged from 23 to 82 m^2^/g with a massive rise in microporosity (5.2 to 62.2 m^2^/g). This opened the zeolite’s 3D channels for continuous uptake, whereas acid-exfoliated bentonite remains a planar 2D surface. HDTMA functionalization exposed the key role of mineral architecture. For bentonite, both single (B-S) and sequential (B-AS) modifications performed poorly, plateauing at 2 mg/g. In contrast, the organozeolites reached 6 mg/g and 14.8 mg/g for C-S and C-AS respectively. This difference occurs because the open 2D layers of bentonite shield its surface, whereas the 3D channels of clinoptilolite allow HDTMA to penetrate and create an organophilic environment that strongly attracts the drug. These results can be supported by FTIR analysis, where the disappearance of water bands (~3400 and ~1640 cm^−1^) and the emergence of HDTMA alkyl bands (2920–2850 cm^−1^) show that the surfactant displaced water to generate hydrophobic binding sites within the zeolite channel [42,43].

The equilibrium adsorption data for bentonite and clinoptilolite modifications were fitted to the Langmuir, Freundlich, and Sips models (Figure 11 and Figure 12; fitting parameters in Table 4). For bentonite, the acid-treated B-A followed the Freundlich model best among the two-parameter models (R^2^ = 0.9329, χ^2^ = 1.844), while Langmuir performed poorly (R^2^ = 0.5362, χ^2^ = 4.387). The three-parameter Sips model achieved a markedly higher correlation coefficient (R^2^ = 0.9971) and a Sips exponent, n_s_ = 3.64, well above unity, confirming the strongly heterogeneous, cooperative character already suggested by the Freundlich fit; however, its χ^2^ (6.62) was actually the highest of the three models, since the Sips curve underestimates q at the lowest Ceq values (where q remains close to zero, inflating the normalized residual). This apparent R^2^/χ^2^ discrepancy simply reflects the sensitivity of χ^2^ to near-zero predicted values rather than a poorer overall fit, and the Freundlich exponent n = 0.613 (<1) still best captures the progressive, cooperative adsorption mechanism on this heterogeneous, exfoliated layered structure [44,45]. In contrast, the surfactant-modified B-S was best described by the Sips model (R^2^ = 0.949, χ^2^ = 0.273), which outperformed both Freundlich (R^2^ = 0.8872, χ^2^ = 0.339) and Langmuir (R^2^ = 0.6975, χ^2^ = 0.309) on both criteria simultaneously. The Sips exponent (n_s_ = 2.11) confirms the heterogeneous character indicated by the Freundlich n (0.672), while the intermediate qmax (2.46 mg g^−1^) reflects that the organophilic transition partially restricts interlayer access, reducing the experimental capacity [46,47]. The dual-modified B-AS was likewise best fitted by Sips (R^2^ = 0.9643, χ^2^ = 0.165), improving on Langmuir (R^2^ = 0.9490, χ^2^ = 0.255) and Freundlich (R^2^ = 0.9575, χ^2^ = 0.196). The high Sips exponent (n_s_ = 3.78), consistent with the Freundlich n = 1.71 (>1), indicates more favorable adsorption energetics than B-S, suggesting the combined treatment partially mitigates adsorbate–adsorbate repulsion. However, this does not translate into higher capacity—the Sips q_max_ (2.07 mg g^−1^) remains one of the lowest among all materials studied (Table 4), reinforcing that limited accessible surface area, not affinity, remains the dominant constraint [48,49].

Clinoptilolite (C) exhibited an excellent fit to the Langmuir isotherm (R^2^ = 0.9942, χ^2^ = 0.233), which remained the best-performing model even after including Sips (R^2^ = 0.9483, χ^2^ = 0.214); the Sips exponent (n_s_ = 1.23) close to unity is itself consistent with the essentially homogeneous, monolayer behavior captured by Langmuir, and the marginal improvement in χ^2^ does not offset the loss in R^2^ from the additional fitting parameter. This indicates that C behaves as a genuinely homogeneous adsorbent for naproxen without needing a more complex model. The acid treatment (C-A), however, led to a reduced Langmuir fit (R^2^ = 0.8267, χ^2^ = 2.382) and a comparatively worse Freundlich fit (R^2^ = 0.9739, χ^2^ = 8.042, the highest χ^2^ recorded for any material/model combination); the Sips model resolved this ambiguity, achieving both the highest R^2^ (0.9804) and the lowest χ^2^ (2.037) of the three models, with n_s_ = 2.45 confirming pronounced surface heterogeneity generated by the removal of occluded species and the opening of channel networks [41,45]. The HDTMA-modified C-S yielded nearly identical Langmuir and Freundlich fits (R^2^ ≈ 0.95, χ^2^ = 0.964 and 0.673, respectively) with n = 1.52; the Sips exponent (n_s_ = 0.70) sits below unity and, together with the fact that qmax could not be uniquely resolved by the nonlinear fit (the data do not reach a clear plateau within the measured Ceq range), indicates that Sips effectively collapses toward Freundlich-type behavior for this material—supporting Freundlich as the more physically interpretable model despite its marginally higher χ^2^ (0.673 vs. 0.568 for the constrained Sips fit) [45,48]. Finally, C-AS fit the Freundlich model slightly better than Langmuir (R^2^ = 0.9892 vs. 0.9576; χ^2^ = 2.625 vs. 1.739), while Sips gave an intermediate result (R^2^ = 0.9846, χ^2^ = 1.987) with n_s_ = 1.25—again indicating that qmax was not fully constrained by the available data (see qmax = 29.74 mg g^−1^, well above the highest measured q of 14.87 mg g^−1^) and should be interpreted with caution. Taken together, the Langmuir, Freundlich, and Sips results converge on the same conclusion: the functionalized surface remains energetically heterogeneous rather than uniform, consistent with the multi-stage, diffusion-influenced behavior seen in the Weber–Morris analysis, and likely reflects organophilic HDTMA domains combined with residual heterogeneity from acid-opened zeolitic microenvironments [36,50].

It is noted that a Freundlich exponent n > 1 indicates favorable, cooperative-type adsorption on a heterogeneous surface, and should not be interpreted as evidence of monolayer coverage, which is instead assessed here via the Sips exponent’s proximity to unity and via direct comparison with the Langmuir fit.

Table 5 compares the naproxen adsorption capacities obtained in this study with those reported for other clay, zeolite, and carbon-based adsorbents. Reported capacities span roughly 6 to 106 mg g^−1^, depending strongly on adsorbent type, synthesis complexity, and adsorption conditions. Within this range, the sequentially modified clinoptilolite (C-AS) developed here (14.8–19 mg g^−1^, or up to 29.7 mg g^−1^ per the Sips-estimated q_max_) performs comparably to, or better than, several other modified natural minerals while requiring only a simple two-step acid/surfactant treatment rather than the specialized synthesis needed for engineered carbons, metal–organic frameworks (MOFs), or covalent organic frameworks that reach the higher end of the range.

#### 2.2.4. Temperature Effect

The effect of temperature on the adsorption equilibrium was evaluated at 25 °C, 30 °C, and 35 °C for the optimized materials (Figure 13). In both systems (C-AS and B-A), the adsorption capacity exhibited a positive thermal dependence, increasing as the temperature was raised, which confirms the endothermic nature of the adsorption process. In the sequential acid–surfactant clinoptilolite (C-AS), the thermal increase enhanced adsorbate partitioning, reaching capacities of 14.6, 16.5, and 19.9 mg g^−1^ at 25, 30, and 35 °C, respectively, for the highest concentrations evaluated. Similarly, the acid-activated bentonite (B-A) exhibited a notable increase in its uptake, from 7.3 mg g^−1^ at 25 °C to 8.7 mg g^−1^ at 30 °C and 10.1 mg g^−1^ at 35 °C. Thermodynamic parameters were derived from the temperature dependence of the standard dimensionless equilibrium constant (*K*°) using the Van’t Hoff equation:

(1) $$\ln K{}^{\circ}=-\frac{\Delta H{}^{\circ}}{\mathrm{RT}}+\frac{\Delta S{}^{\circ}}{R}$$

(2) ΔG°=ΔH°−TΔS°

where ΔH° (kJ mol^−1^) and ΔS° (J mol^−1^ K^−1^) were obtained from the slope and intercept of the ln(K°) vs. 1/T plot, respectively, R (8.314 J mol^−1^ K^−1^) is the universal gas constant, and T (K) is the absolute temperature.

The resulting parameters are summarized in Table 6. For C-AS, the calculated enthalpy change ΔH° was 35.28 kJ mol^−1^, whereas B-A yielded a ΔH° of 32.25 kJ mol^−1^. Both values fall within the typical range for physisorption involving hydrophobic and van der Waals interactions, and the positive sign confirms the endothermicity of the uptake [45]. Additionally, the positive entropy changes (ΔS° = 134.93 J mol^−1^ K^−1^ for C-AS and 116.14 J mol^−1^ K^−1^ for B-A) indicate a significant increase in randomness at the solid–liquid interface during adsorption. This entropic gain is primarily attributed to the release of solvation water molecules from the hydrated adsorbate and the mineral surface, which are displaced into the bulk solution upon naproxen uptake [47]. The negative ΔG° values obtained (−4.95, −5.63, and −6.30 kJ mol^−1^ for C-AS at 25, 30, and 35 °C, respectively; −2.38, −2.96, and −3.54 kJ mol^−1^ for B-A at the same temperatures) confirm the spontaneous nature of the adsorption process. These negative values fall within the typical range for physisorption (−20 to 0 kJ mol^−1^), consistent with the proposed hydrophobic and van der Waals interactions. The more negative ΔG° values at higher temperatures indicate enhanced spontaneity, in agreement with the endothermic nature of the process (ΔH° > 0) [38].

#### 2.2.5. Proposed Adsorption Mechanism

For bentonite-based materials XRD and FTIR confirm that the montmorillonite framework remains structurally intact across all treatments, with only a slight (001) shift toward lower 2θ in the HDTMA-modified samples (B-S, B-AS), consistent with surfactant intercalation expanding the interlayer basal spacing rather than altering the layered structure. Acid activation (B-A) produced only modest textural gains (BET area, microporosity, and pore volume increases of ≤42% relative to natural B), since HCl treatment on bentonite mainly leaches octahedral cations and removes impurities without generating substantial new porosity in its 2D layered architecture [1,3]. Consequently, HDTMA modification is most effective when applied directly to the untreated mineral (B-S), which showed the largest textural gains (204% increase in BET area), consistent with surfactant-driven interlayer expansion acting on the still-compact native structure [4]. When acid activation precedes HDTMA loading (B-AS), this textural gain is markedly reduced, indicating that the surfactant re-occupies porosity already altered by acid pretreatment rather than acting synergistically with it. This structural picture directly explains the observed kinetic and equilibrium behavior: B-A, governed by slow, swelling-driven diffusion into acid-exfoliated interlayer galleries (lowest PSO and Avrami rate constants; Table 2 and Table 3), achieves the highest bentonite capacity despite its slower uptake, while the HDTMA-modified bentonites (B-S, B-AS) saturate rapidly but at comparatively low capacity, consistent with adsorption confined largely to the external, organophilic surface rather than the interlayer space.

For the clinoptilolite series, the natural sample (C) exhibits hierarchical porosity arising from its zeolitic channel system, combining microporous and mesoporous contributions associated with intercrystalline and interparticle spaces [6]. Acid activation (C-A) produced a substantial increase in BET surface area and, especially, microporosity (258% and 1090% relative to natural C, respectively). This textural response is consistent with, though not directly confirmed by, dealumination-driven clearing of extra-framework cations and channel opening, a mechanism well documented for acid-treated clinoptilolite in the literature [7]. Because this channel system remains structurally intact after acid treatment, subsequent HDTMA loading in C-AS is proposed to penetrate and functionalize the newly accessible microporosity, plausibly generating organophilic domains distributed through the particle rather than confined to the external surface, consistent with the kinetic and capacity trends observed contrasting to the interlayer-blocking behavior observed for HDTMA on bentonite. This acid–surfactant synergy, unique to the rigid clinoptilolite framework, accounts for the prolonged, diffusion-influenced kinetics observed for C-AS, reflected in its markedly sub-unitary Avrami exponent and its multilinear Weber–Morris profile, as well as its highest overall adsorption capacity among all eight materials studied.

Although desorption was beyond the scope of this study, the literature supports the regenerability of comparable acid-activated, surfactant-modified adsorbents. Reversible NSAID adsorption/desorption on HDTMA-bentonite has been reported [55], as well as multi-cycle regeneration of organoclays for other organic pollutants, albeit with a gradual efficiency decline [56,57]. Similar cycle-dependent losses occur in zeolite adsorbents regenerated via advanced oxidation [58]. Based on these precedents and the observed pH-dependence, pH-shift elution is proposed as the most promising strategy for future evaluation.

A limitation of this study is that the same HDTMA concentration was applied to both minerals without independently determining their cation-exchange capacities (CEC), which may differ substantially. FTIR confirmed surfactant anchoring on both materials (Section 2.1), but the fraction of exchange sites occupied may not be directly comparable between series. Quantifying CEC and HDTMA loading is identified as a priority for future work.

## 3. Materials and Methods

### 3.1. Acid Activation and Surfactant Modification

Natural bentonite (B) and natural clinoptilolite (C) were first purified through acid activation. Briefly, 50 g of each clay was dispersed in 600 mL of 6 M HCl and stirred magnetically at 300 rpm for 4 h at 60 °C. The resulting suspension was vacuum filtered through Whatman No. 40 filter paper, and the solid retained was repeatedly washed with hot distilled water until the filtrate tested negative for chloride ions, as verified by the AgNO_3_ test. The purified solids were then oven-dried at 105 °C overnight. The acid-activated products derived from bentonite and clinoptilolite were designated BA and CA, respectively.

The natural and acid-treated clays (B, BA, C, and CA) were subsequently modified with hexadecyltrimethylammonium bromide (HDTMA) to introduce organophilic character to their surfaces. For each modification, 30 g of clay was added to 500 mL of an aqueous HDTMA solution at a concentration of 1000 mg L^−1^, and the suspension was stirred at 300 rpm for 4 h at 60 °C. The modified solids were separated by filtration and washed repeatedly with hot distilled water until the supernatant showed no residual bromide, as confirmed by the AgNO_3_ test. The materials were then dried overnight at 105 °C, ground, and sieved through a No. 65 mesh to obtain a homogeneous particle size. All samples were stored in airtight containers until further use. The HDTMA-modified materials were labeled B-S, B-AS, C-S, and C-AS, corresponding to the surfactant-modified natural bentonite, acid-activated bentonite, natural clinoptilolite, and acid-activated clinoptilolite, respectively. See Figure 14.

### 3.2. Characterization Techniques

The mineralogical, chemical, and textural properties of the natural, acid-treated, and HDTMA-modified materials were evaluated using a combination of complementary techniques. Mineralogical composition was determined by X-ray diffraction (XRD) on a PANalytical Empyrean diffractometer (Almelo, The Netherlands) equipped with a Cu Kα radiation source, scanning over a 2θ range of 5–90° with a step size of 0.020° and a counting time of 4 s per step at 25 °C. The surface functional groups of the materials were identified by Fourier-transform infrared spectroscopy (FTIR) using a PerkinElmer Frontier spectrophotometer (Waltham, MA, USA) fitted with an attenuated total reflectance (ATR) accessory. Spectra were recorded over 64 scans at a resolution of 4 cm^−1^. Textural properties, including specific surface area and pore-size distribution, were determined by nitrogen physisorption using a NOVA Touch LX1 gas sorption analyzer (Quantachrome Instruments, Boynton Beach, FL, USA).

### 3.3. Adsorption Studies

The adsorption behavior of naproxen onto the natural, acid-treated, and HDTMA-modified materials was evaluated through pH-dependent, kinetic, and equilibrium studies. Adsorption experiments were first carried out at four pH values (4, 6, 8 and 10) using an initial naproxen concentration of 100 mg/L in 20 mL of solution, at 25 °C and a contact time of 24 h, to identify the pH at which adsorption capacity was maximized. This optimum pH was then used for all subsequent kinetic and equilibrium experiments.

Adsorption kinetics were investigated over contact times ranging from 1 to 240 min at the optimum pH. The experimental data were fitted to the pseudo-first-order (PFO), pseudo-second-order (PSO), and Weber–Morris intraparticle diffusion models to describe the rate and mechanism of naproxen uptake over time. Adsorption isotherms were obtained at the optimum pH over a naproxen concentration range of 1–500 mg/L, at temperatures of 25, 30 and 35 °C and a fixed contact time of 24 h using 20 mL of solution. The equilibrium data were analyzed using the Langmuir, Freundlich and Sips models to characterize the adsorption capacity and the nature of the adsorbate–adsorbent interactions at each temperature.

For all experiments, 20 mg of adsorbent was added to the corresponding naproxen solution, and the suspensions were stirred for the specified contact time. After equilibration, the suspensions were centrifuged to separate the solid phase, and the residual naproxen concentration in the supernatant was determined by ultraviolet–visible (UV–Vis) spectroscopy at 272 nm using a Thermo Scientific Genesys 180 spectrophotometer (Waltham, MA, USA). The amount of naproxen adsorbed at equilibrium, q (mg/g), was calculated as

(3) $$q=\left(C_{0}-C\right)\frac{V}{m}$$

where C_0_ and C (mg/L) are the initial and residual naproxen concentrations, respectively, V (L) is the volume of solution, and m (g) is the mass of adsorbent used. All batch adsorption experiments (kinetics, equilibrium isotherms, pH, and temperature studies) were performed in duplicate (n = 2), and the reported values represent the mean of the two independent determinations. Error bars shown in the corresponding figures represent the standard deviation between duplicates.

In all cases, the kinetic and isotherm parameters were estimated by nonlinear regression using the Generalized Reduced Gradient (GRG) nonlinear algorithm.

The pseudo-first-order (PFO) and pseudo-second-order (PSO) kinetic models were applied in their nonlinear forms:

(4) $$q_{t}=q_{e}\left(1-e^{-k_{1}t}\right)$$

(5) $$q_{t}=\frac{k_{2}q_{e}^{2}t}{1+k_{2}q_{e}t}$$

where q_t_ and q_e_ (mg g^−1^) are the amounts of naproxen adsorbed at time t and at equilibrium, respectively, and k_1_ (min^−1^) and k_2_ (g mg^−1^ min^−1^) are the corresponding rate constants. To complement these integer-order models, the data were additionally fitted to the Elovich and Avrami fractional-order kinetic models. The Elovich equation, in its non-singular integrated form, was used as

(6) $$q_{t}=\frac{1}{\beta }\ln\left(1+\mathrm{\alpha \beta t}\right)$$

where α (mg g^−1^ min^−1^) is the initial adsorption rate and β (g mg^−1^) is related to the extent of surface coverage and activation energy for chemisorption [34]. The Avrami fractional-order model was applied as

(7) $$q_{t}=q_{e}\left[1-\exp\left(-{\left(k_{\mathrm{AV}}\;t\right)}^{n_{\mathrm{AV}}}\right)\right]$$

where k_AV_ (min^−1^) is the Avrami rate constant and n_AV_ is a fractional exponent related to the number of adsorption mechanisms or pathways contributing to the overall process [36].

The Weber–Morris intraparticle diffusion model was used to evaluate the contribution of diffusional mechanisms to the overall adsorption rate:

(8) $$q_{t}=k_{\mathrm{id}}t^{0.5}+C$$

where k_id_ (mg g^−1^ min^−0.5^) is the intraparticle diffusion rate constant and C (mg g^−1^) is the interception.

Equilibrium data were fitted to the Langmuir, Freundlich and Sips isotherm models:

(9) $$q_{e}=\frac{Q_{\max}K_{L}C_{e}}{1+K_{L}C_{e}}$$

(10) $$q_{e}=K_{f}C_{e}^{1/n}$$

(11) $$q_{e}=\frac{q_{\max}{{(K}_{s}C_{e})}^{n}\;}{1+{{(K}_{s}C_{e})}^{n}}$$

where Q_max_ (mg g^−1^) is the maximum monolayer adsorption capacity, K_L_ (L mg^−1^) is the Langmuir affinity constant, C_e_ (mg L^−1^) is the equilibrium naproxen concentration, and K_f_ ((mg g^−1^)(L mg^−1^)^1/n^) and n are the Freundlich capacity and heterogeneity parameters, respectively. Also, for the Sips model in Equation (9), q_max_ (mg g^−1^) is the maximum adsorption capacity, K_S_ (L mg^−1^)^nS^ is the Sips affinity constant and n_S_ is the Sips heterogeneity exponent.

### 3.4. Use of Generative Artificial Intelligence

During the preparation of this work, the authors used Claude Sonnet 5.5 (Anthropic, San Francisco, CA, USA) to support editorial review of the manuscript, to help identify potential methodological inconsistencies in the text, and to assist in the production of the graphical abstract. All experimental work, data acquisition, and scientific interpretation were performed by the authors. The authors reviewed and edited all AI-assisted content and take full responsibility for the content of this publication.

## 4. Conclusions

This study demonstrates that the effectiveness of acid-activation and HDTMA modification for naproxen removal is strongly governed by the structural architecture of the host mineral rather than by the treatment sequence itself. XRD and FTIR confirmed that both acid activation and surfactant loading preserved the crystalline frameworks of bentonite and clinoptilolite, while textural analysis revealed contrasting responses: acid treatment produced the greatest gains for clinoptilolite through dealumination and channel opening, whereas HDTMA modification alone produced the largest textural development in bentonite through interlayer expansion. This combined treatment was synergistic for clinoptilolite, where acid-opened channels enabled deeper HDTMA penetration and the highest overall capacity, but not for bentonite, where surfactant loading partially re-occupied the porosity generated by acid activation rather than acting synergistically with it.

This structural divergence translated directly into adsorption performance. For bentonite, acid-activated B-A was the best-performing material, reaching an equilibrium capacity of approximately 7.3 mg g^−1^, while surfactant-modified samples (B-S, B-AS) showed rapid but limited uptake due to pore blockage within the expanded interlayer galleries. For clinoptilolite, the sequentially modified C-AS markedly outperformed all other materials, reaching 14.8, 16.5, and 19.9 mg g^−1^ at 25, 30, and 35 °C, respectively, benefiting from the combined effect of acid-induced channel accessibility and HDTMA-derived organophilicity. Kinetic data were best described by the pseudo-second-order model for most modified materials, while the Elovich and Avrami fractional-order models provided superior fits overall, with sub-unitary Avrami exponents pointing to a heterogeneous, multi-pathway adsorption mechanism rather than a single elementary step; Weber–Morris analysis confirmed that intraparticle diffusion was not the sole rate-controlling step in any system. Equilibrium behavior followed mixed Langmuir–Freundlich trends that were resolved using the Sips model, reflecting the heterogeneous nature of the modified surfaces, and thermodynamic parameters derived from a three-temperature (25–35 °C) study confirmed spontaneous, endothermic adsorption consistent with a physisorption-dominated mechanism for both optimized materials (C-AS and B-A). Overall, these results indicate that zeolitic frameworks benefit more from combined acid–surfactant functionalization than layered clays, positioning acid-activated and HDTMA-modified clinoptilolite as a promising, low-cost adsorbent for naproxen removal from water, while highlighting that treatment strategies for pharmaceutical adsorption should be tailored to the specific mineral architecture rather than applied uniformly across adsorbent classes. Future work should address adsorbent regeneration and reuse cycling with post-adsorption characterization, quantification of cation-exchange capacity and HDTMA loading per mineral, compositional analysis (e.g., Si/Al ratio via X-ray fluorescence or energy-dispersive X-ray spectroscopy) to confirm the proposed dealumination mechanism, and molecular-level (DFT) studies to resolve the specific naproxen–surface interaction mechanisms proposed here.

## Figures and Tables

**Figure 1:**
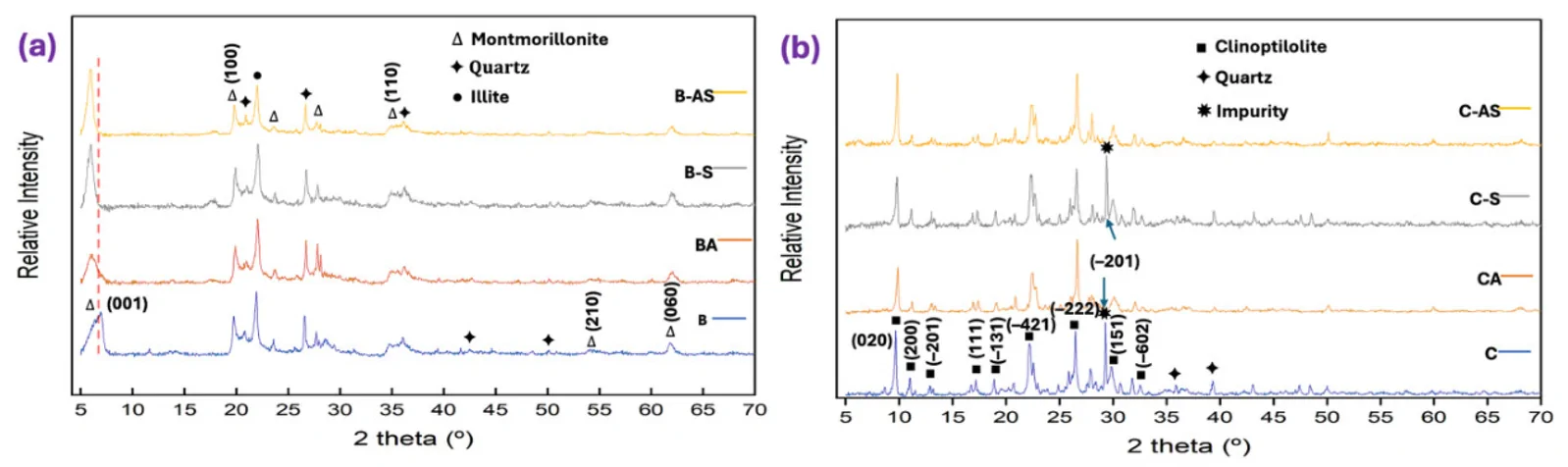
X-ray diffraction (XRD) patterns of natural and modified (**a**) bentonite and (**b**) clinoptilolite, recorded using Cu Kα radiation. B and C denote the natural minerals; BA and CA denote acid-activated materials; and the suffixes -S and -AS indicate HDTMA modification of the natural and acid-activated minerals, respectively. Selected reflections are labeled with phase identifiers and Miller indices. HDTMA: hexadecyltrimethylammonium bromide. The dashed red line marks the position of the (001) basal reflection in the untreated bentonite (B), included as a visual reference to highlight its shift toward lower 2θ values in the HDTMA-modified samples.

**Figure 2:**
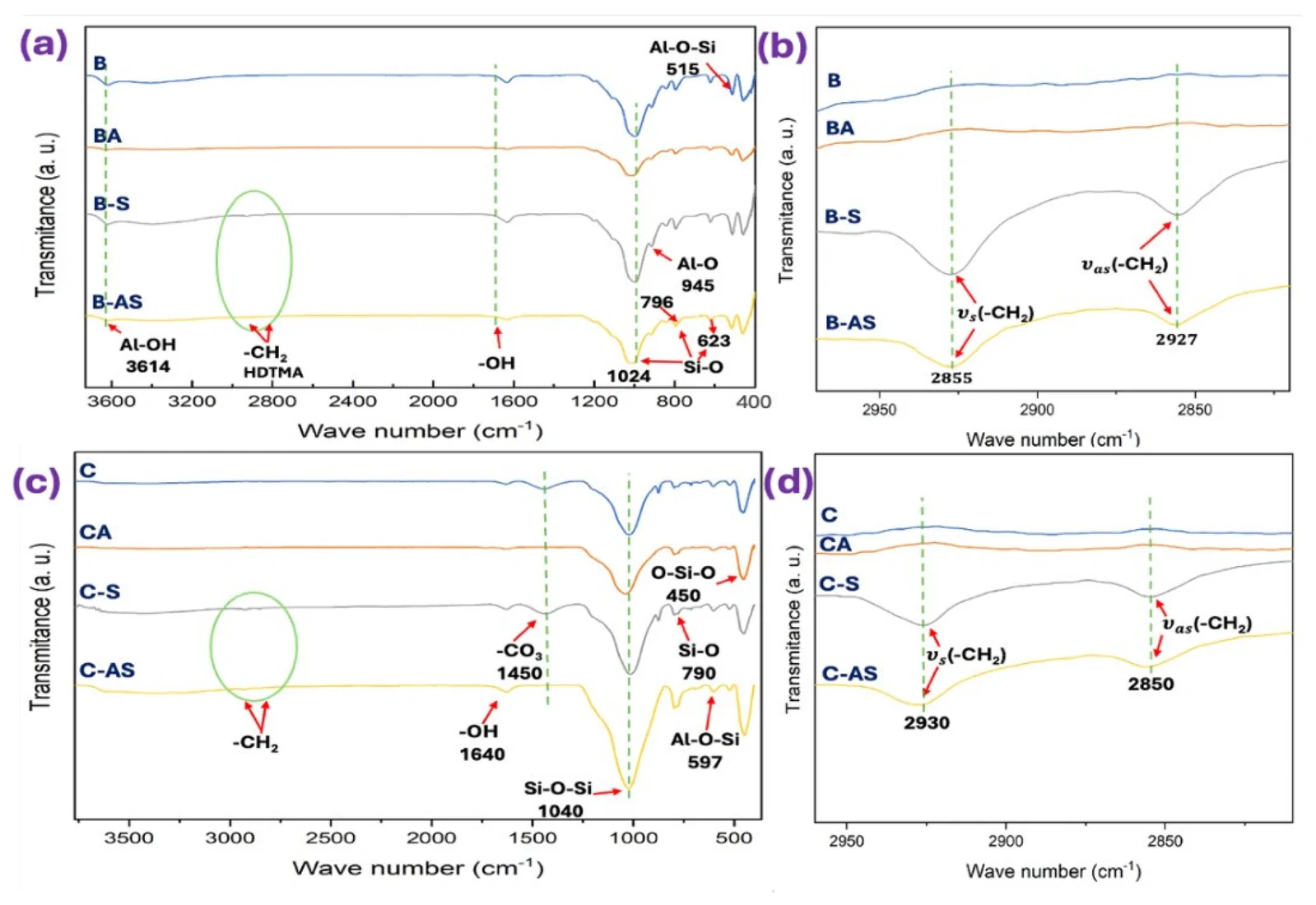
Attenuated total reflectance Fourier-transform infrared (ATR–FTIR) spectra of natural and modified (**a**) bentonite and (**c**) clinoptilolite, with enlarged views of the C–H stretching region for (**b**) bentonite-based materials (2950–2830 cm^−1^) and (**d**) clinoptilolite-based materials (2960–2820 cm^−1^). B and C denote the natural minerals; BA and CA denote acid-activated materials; and the suffixes -S and -AS indicate HDTMA modification of the natural and acid-activated minerals, respectively. HDTMA: hexadecyltrimethylammonium bromide. The green oval in panels (**a**,**c**) highlights the 2800–2950 cm^−^^1^ region where the weak C–H stretching bands of the HDTMA alkyl chains appear, shown magnified in panels (**b**,**d**).

**Figure 3:**
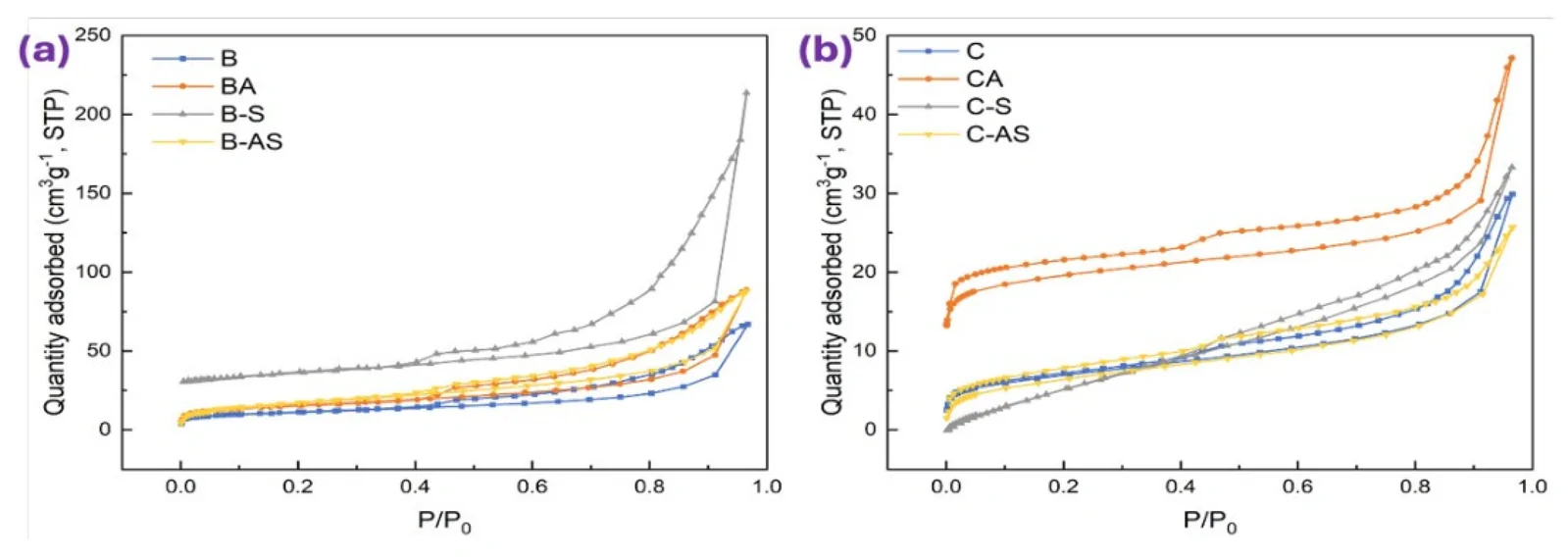
Nitrogen adsorption–desorption isotherms of natural and modified (**a**) bentonite and (**b**) clinoptilolite, showing the adsorbed gas volume at standard temperature and pressure (STP) as a function of relative pressure (P/P_0_). B and C denote the natural minerals; BA and CA denote acid-activated materials; and the suffixes -S and -AS indicate HDTMA modification of the natural and acid-activated minerals, respectively. HDTMA: hexadecyltrimethylammonium bromide.

**Figure 4:**
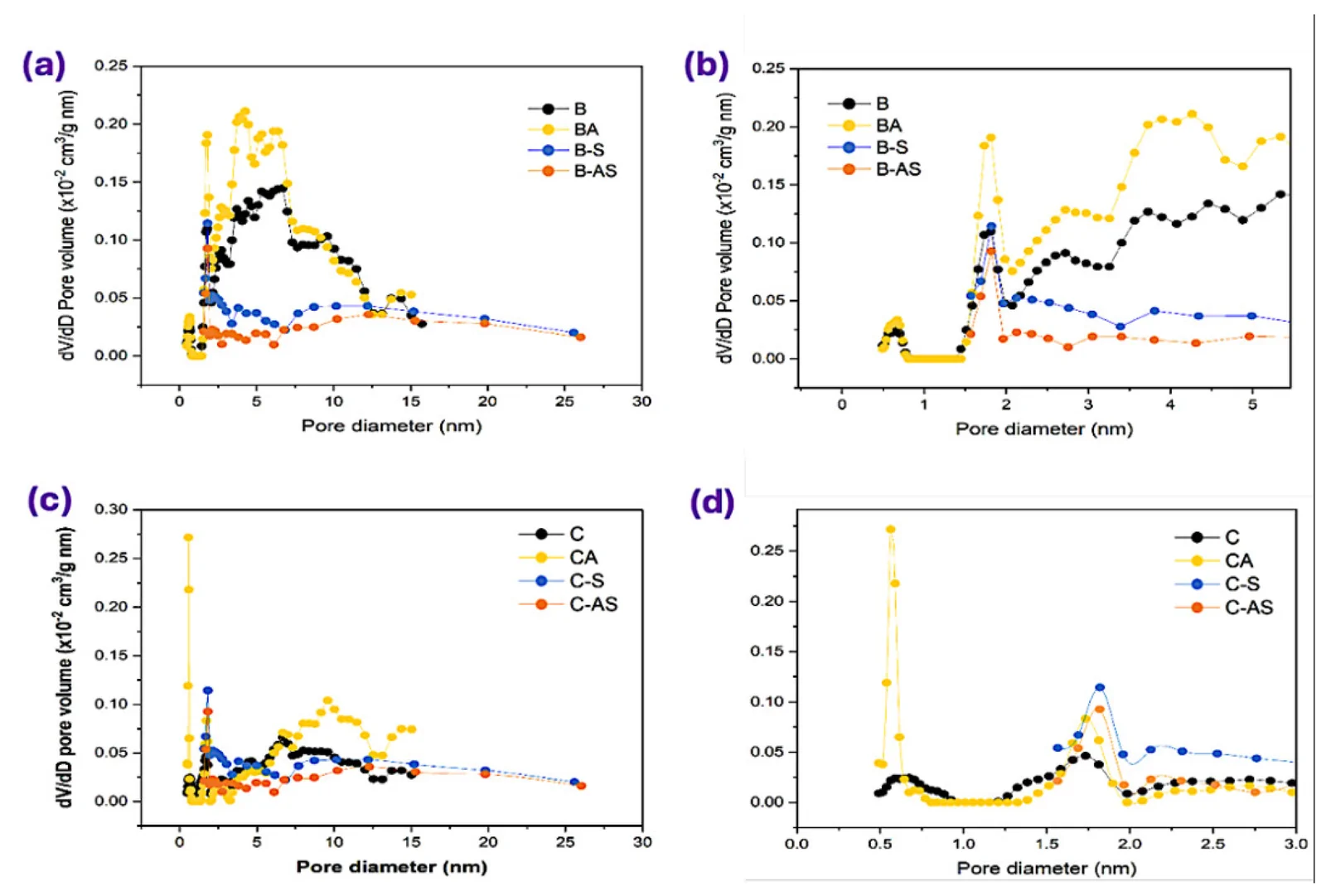
Pore-size distributions (0.5–30 nm) of natural and modified (**a**,**b**) bentonite and (**c**,**d**) clinoptilolite, derived from the nitrogen desorption branch in isotherms in Figure 3. B and C denote the natural minerals; BA and CA denote acid-activated materials; and the suffixes -S and -AS indicate HDTMA modification of the natural and acid-activated minerals, respectively. HDTMA: hexadecyltrimethylammonium bromide.

**Figure 5:**
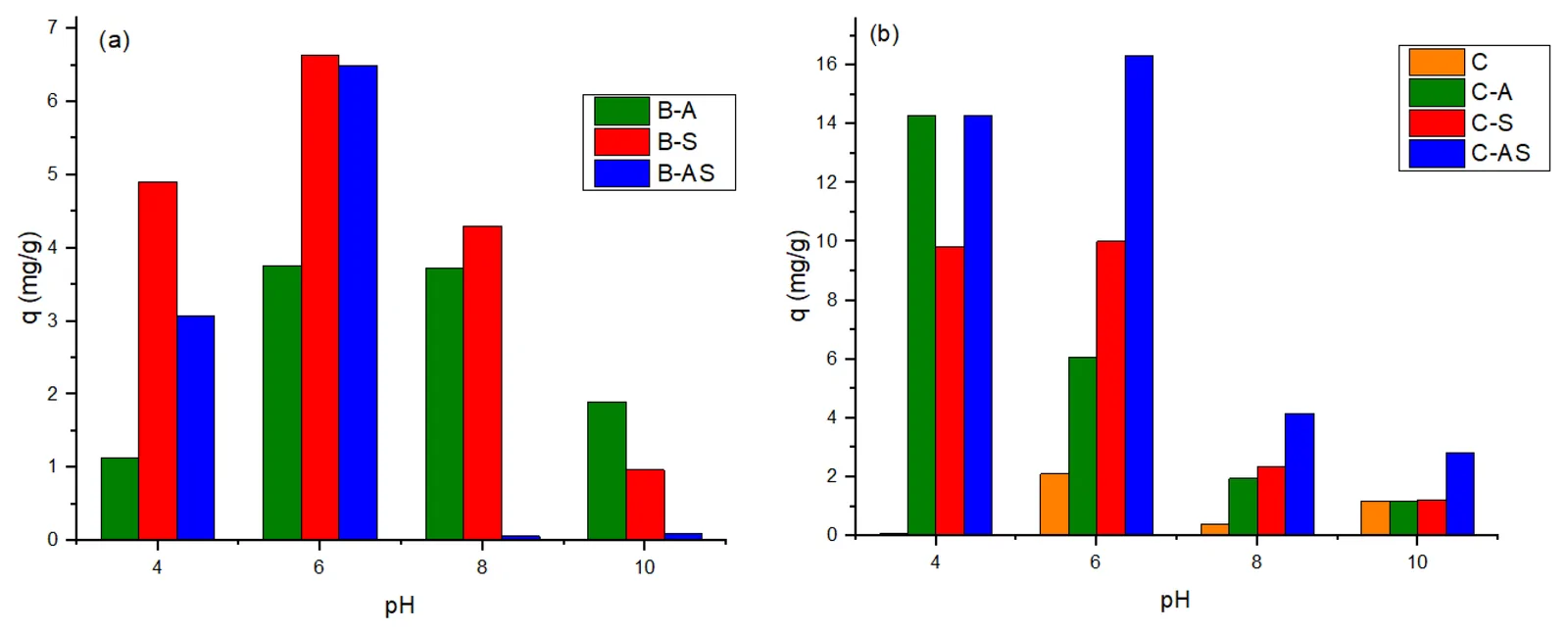
Effect of solution pH on naproxen adsorption capacity (q) for (**a**) bentonite-based and (**b**) clinoptilolite-based materials (C_0_ = 100 mg L^−1^, adsorbent mass = 20 mg, t = 24 h, T = 25 °C). B and C denote bentonite and clinoptilolite, respectively; -A, -S, and -AS indicate acid activation, HDTMA modification, and sequential acid-activation and HDTMA modification, respectively. HDTMA: hexadecyltrimethylammonium bromide.

**Figure 6:**
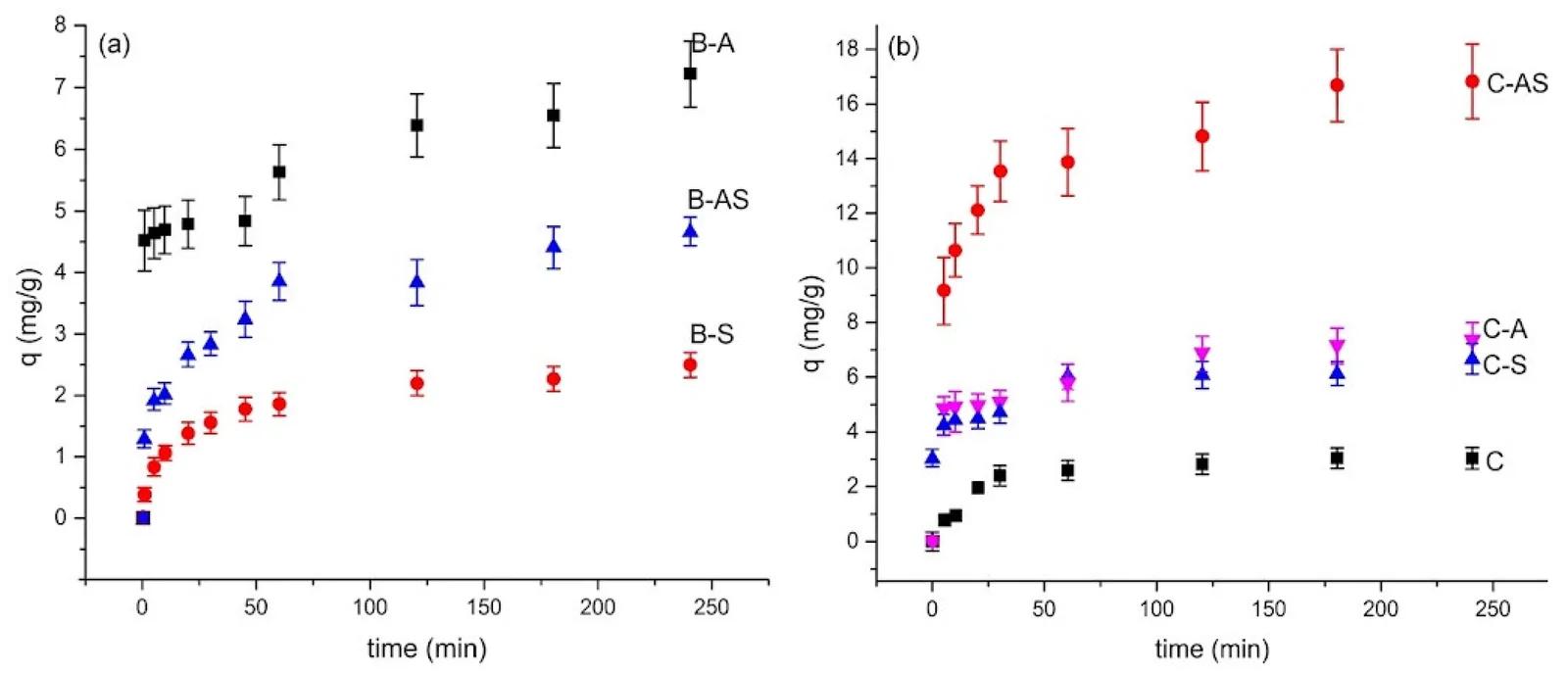
Naproxen adsorption kinetics for (**a**) bentonite-based and (**b**) clinoptilolite-based materials (C_0_ = 100 mg L^−1^, pH = 6, T = 25 °C, adsorbent mass = 20 mg). Symbols represent experimental adsorption capacities at time t (q_t_). B and C denote bentonite and clinoptilolite, respectively; -A, -S, and -AS indicate acid activation, HDTMA modification, and sequential acid-activation and HDTMA modification, respectively. HDTMA: hexadecyltrimethylammonium bromide.

**Figure 7:**
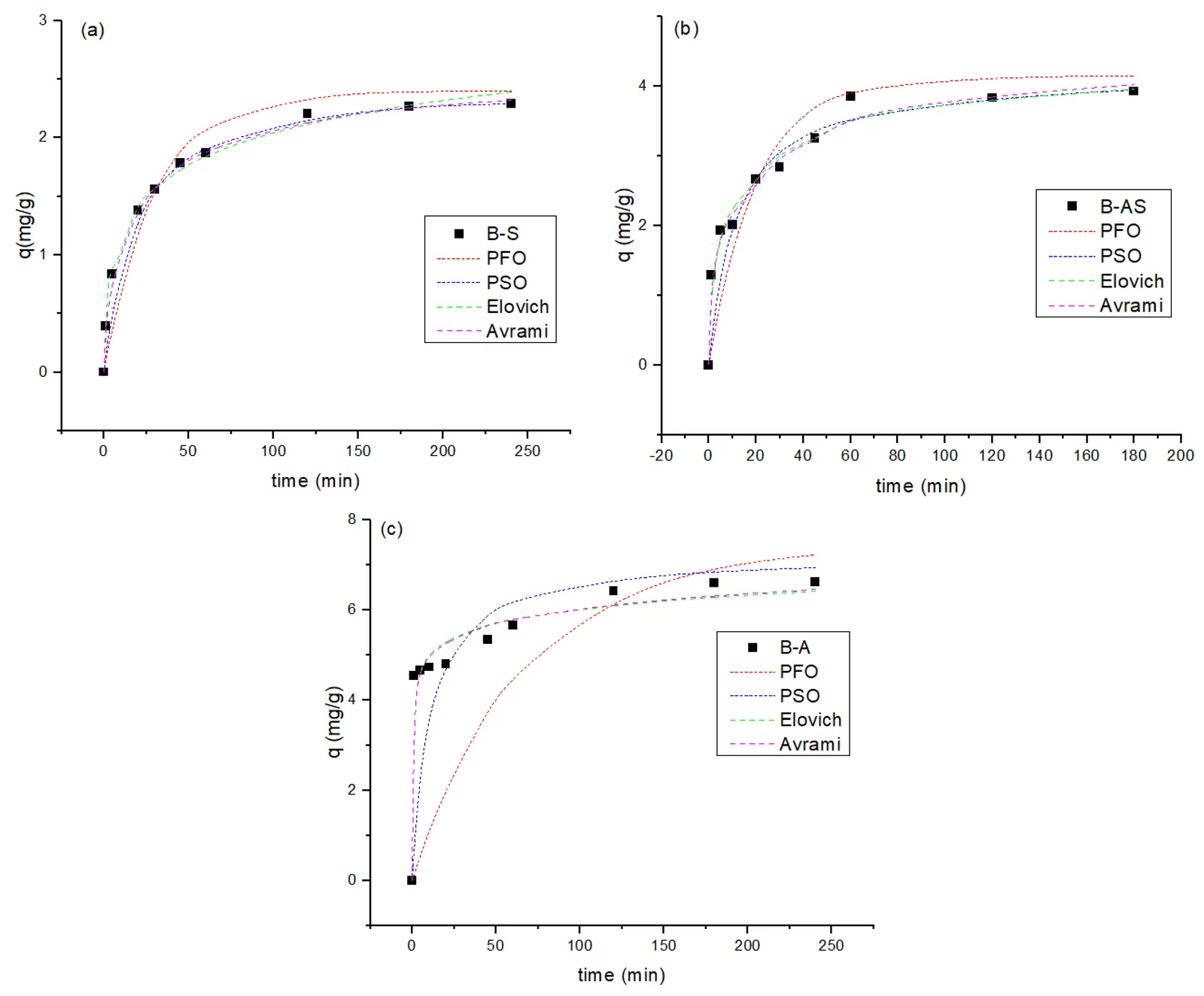
Pseudo-first-order (PFO) and pseudo-second-order (PSO) kinetic models to naproxen adsorption data for (**a**) HDTMA-modified bentonite (B-S), (**b**) acid-activated and subsequently HDTMA-modified bentonite (B-AS), and (**c**) acid-activated bentonite (B-A). Black squares represent experimental data; red, blue, green and magenta dotted lines indicate PFO, PSO, Elovich and Avrami fits, respectively (pH = 6, T = 25 °C, adsorbent mass = 20 mg). HDTMA: hexadecyltrimethylammonium bromide.

**Figure 8:**
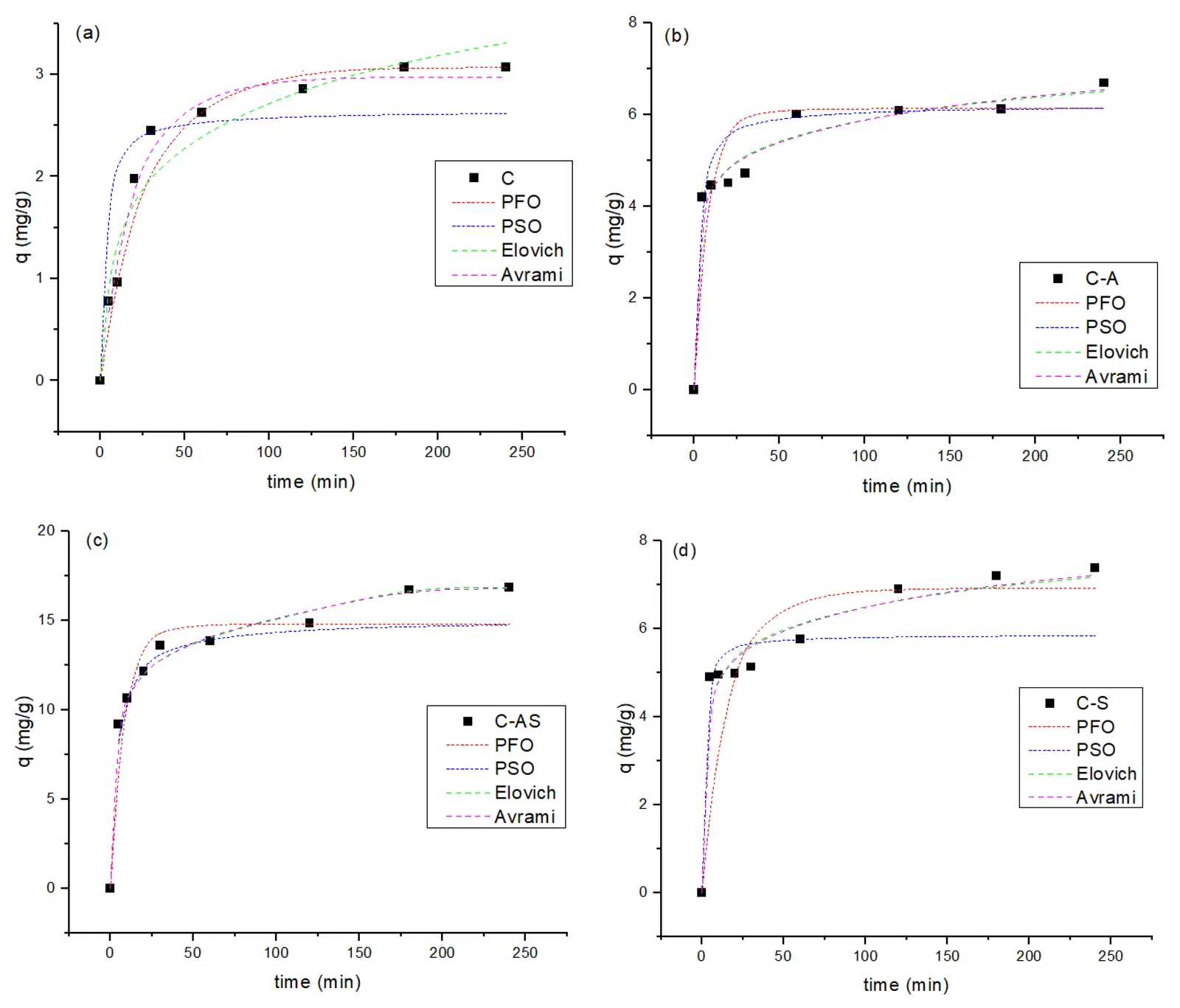
Nonlinear fits of the pseudo-first-order (PFO) and pseudo-second-order (PSO) kinetic models to naproxen adsorption data for (**a**) natural clinoptilolite (C), (**b**) acid-activated clinoptilolite (C-A), (**c**) acid-activated and subsequently HDTMA-modified clinoptilolite (C-AS), and (**d**) HDTMA-modified clinoptilolite (C-S). Black squares represent experimental data; red, blue, green and magenta dotted lines indicate PFO, PSO, Elovich and Avrami fits, respectively (pH = 6, T = 25 °C, adsorbent mass = 20 mg). HDTMA: hexadecyltrimethylammonium bromide.

**Figure 9:**
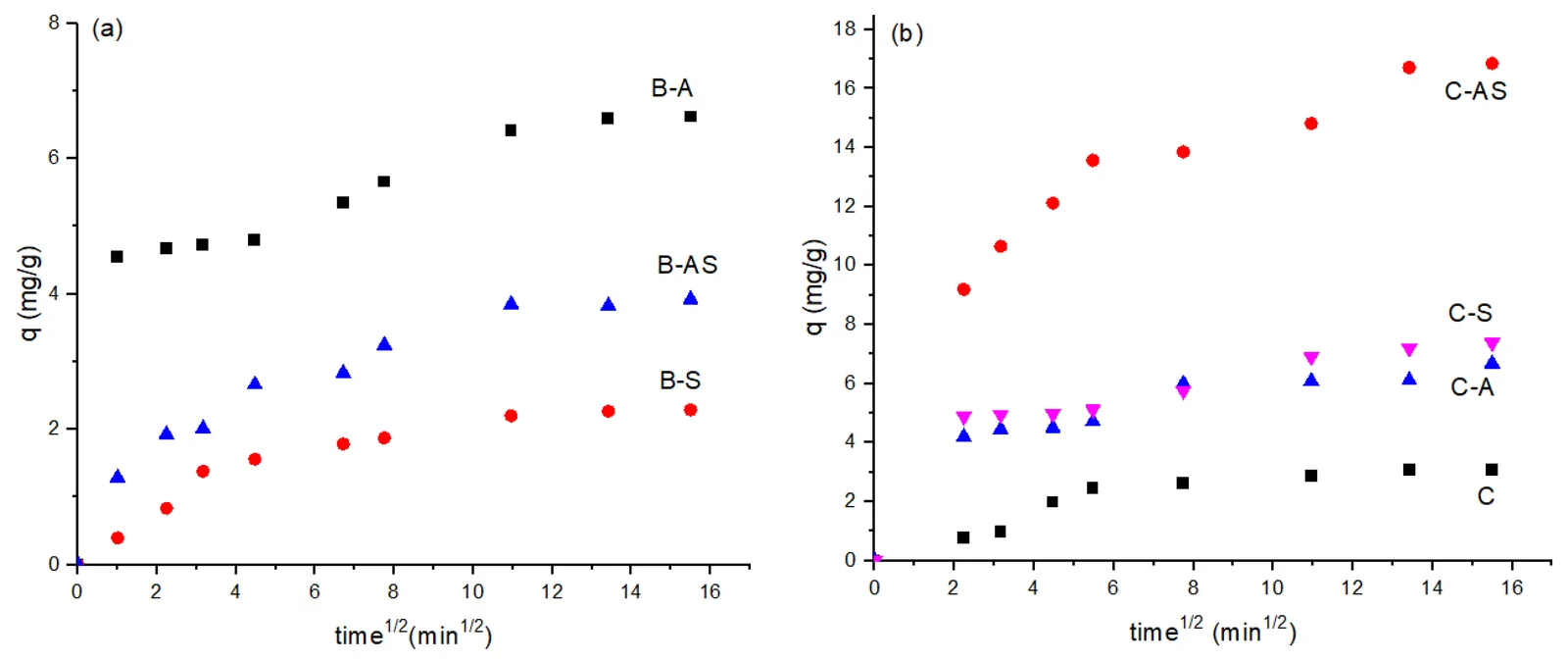
Weber–Morris intraparticle diffusion plots of naproxen adsorption capacity (q) versus the square root of contact time (t^1^ᐟ^2^) for (**a**) bentonite-based and (**b**) clinoptilolite-based adsorbents (pH = 6, T = 25 °C, adsorbent mass = 20 mg). Symbols represent experimental data; linear segmentation was avoided in the figure to prevent the visual implication of distinct transport mechanisms. B and C denote bentonite and clinoptilolite, respectively; -A, -S, and -AS indicate acid activation, HDTMA modification, and sequential acid-activation and HDTMA modification, respectively. HDTMA: hexadecyltrimethylammonium bromide.

**Figure 10:**
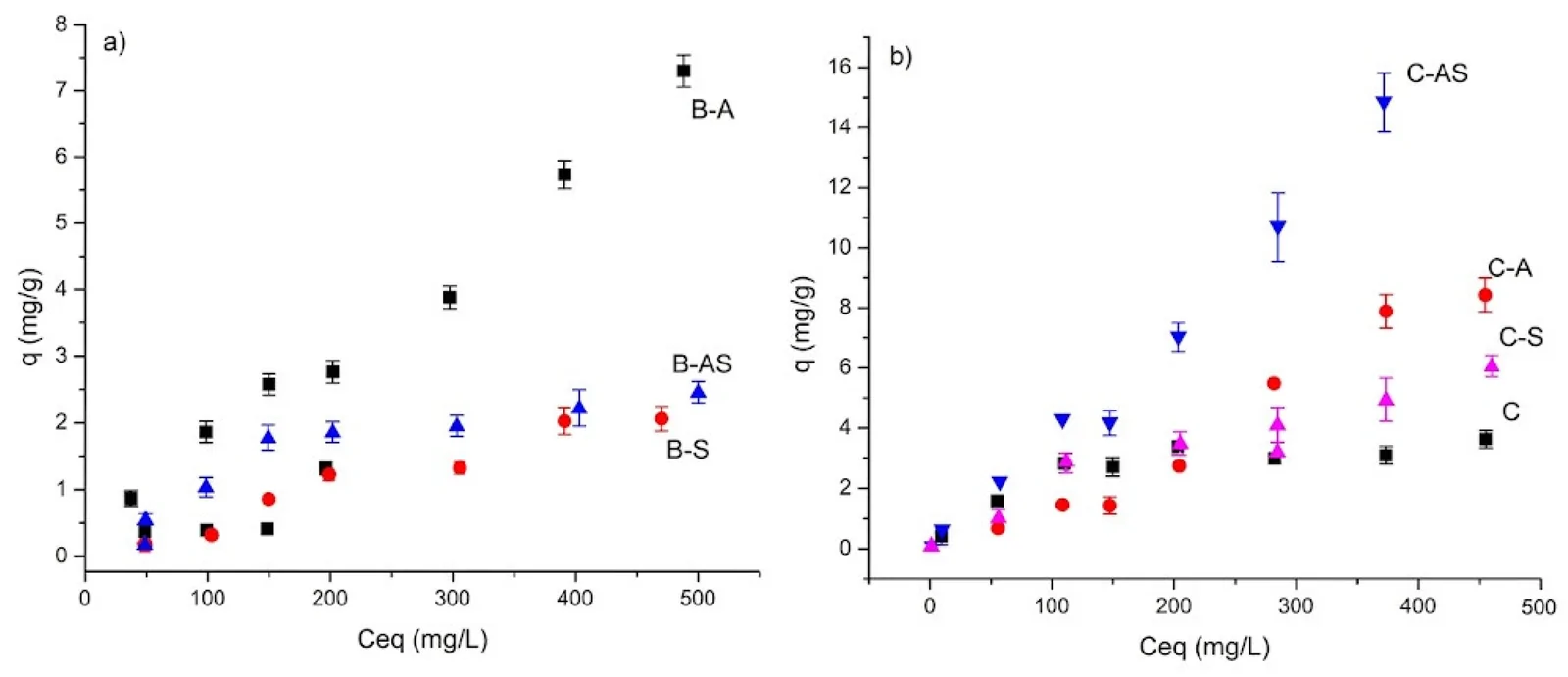
Equilibrium adsorption isotherms of naproxen for (**a**) bentonite-based and (**b**) clinoptilolite-based adsorbents (pH = 6, contact time = 24 h, 25 °C, adsorbent mass = 20 mg). Symbols represent experimental equilibrium adsorption capacities (q_e_) as a function of equilibrium naproxen concentration (C_e_). B and C denote bentonite and clinoptilolite, respectively; -A, -S, and -AS indicate acid activation, HDTMA modification, and sequential acid-activation and HDTMA modification, respectively. HDTMA: hexadecyltrimethylammonium bromide.

**Figure 11:**
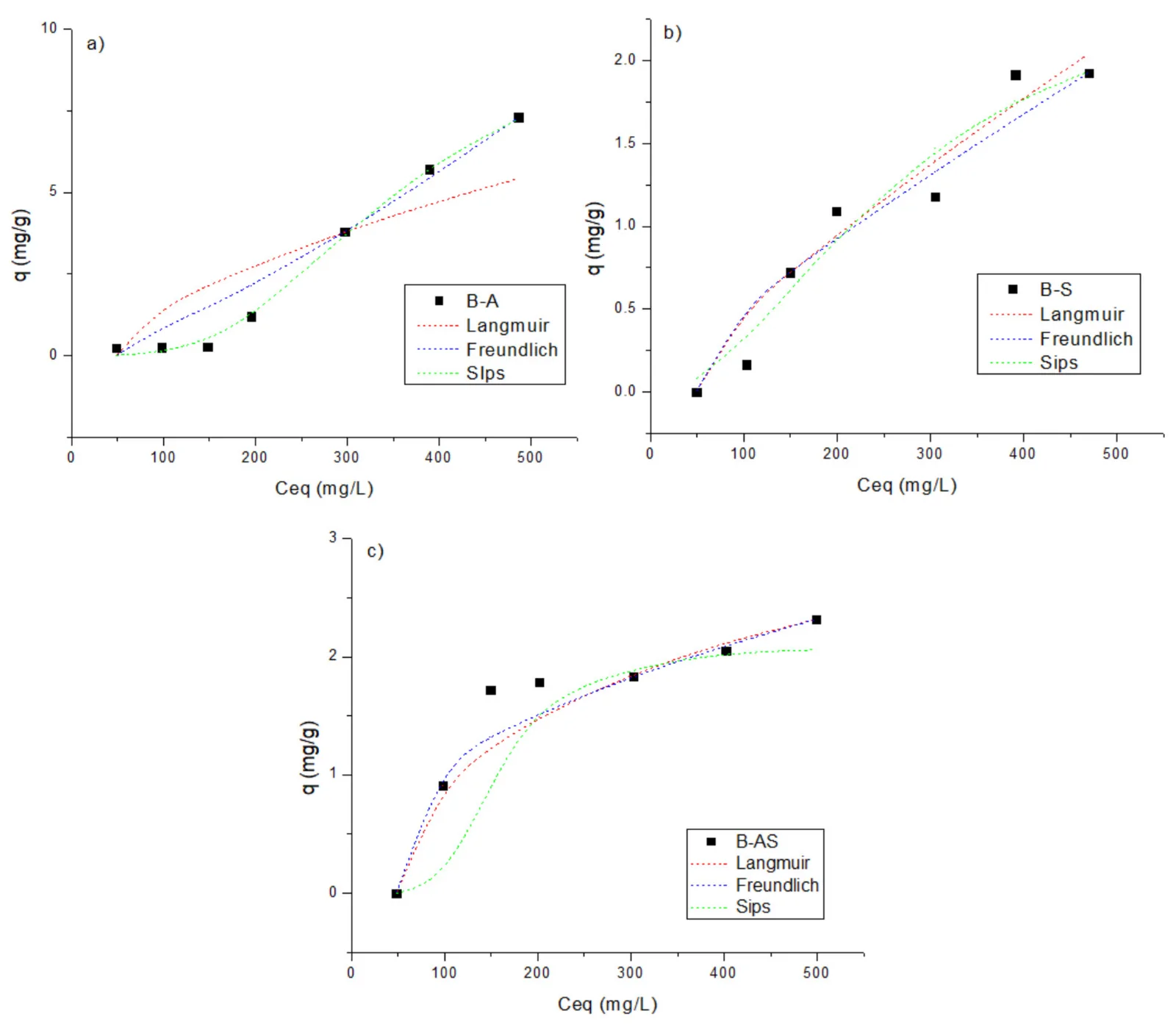
Nonlinear fits of the Langmuir, Freundlich and Sips isotherm models to naproxen equilibrium adsorption data for (**a**) acid-activated bentonite (B-A), (**b**) HDTMA-modified bentonite (B-S), and (**c**) acid-activated and subsequently HDTMA-modified bentonite (B-AS). Black squares represent experimental data; red, blue and green lines indicate Langmuir and Freundlich fits, respectively (pH = 6, 25 °C, contact time = 24 h, adsorbent mass = 20 mg). HDTMA: hexadecyltrimethylammonium bromide.

**Figure 12:**
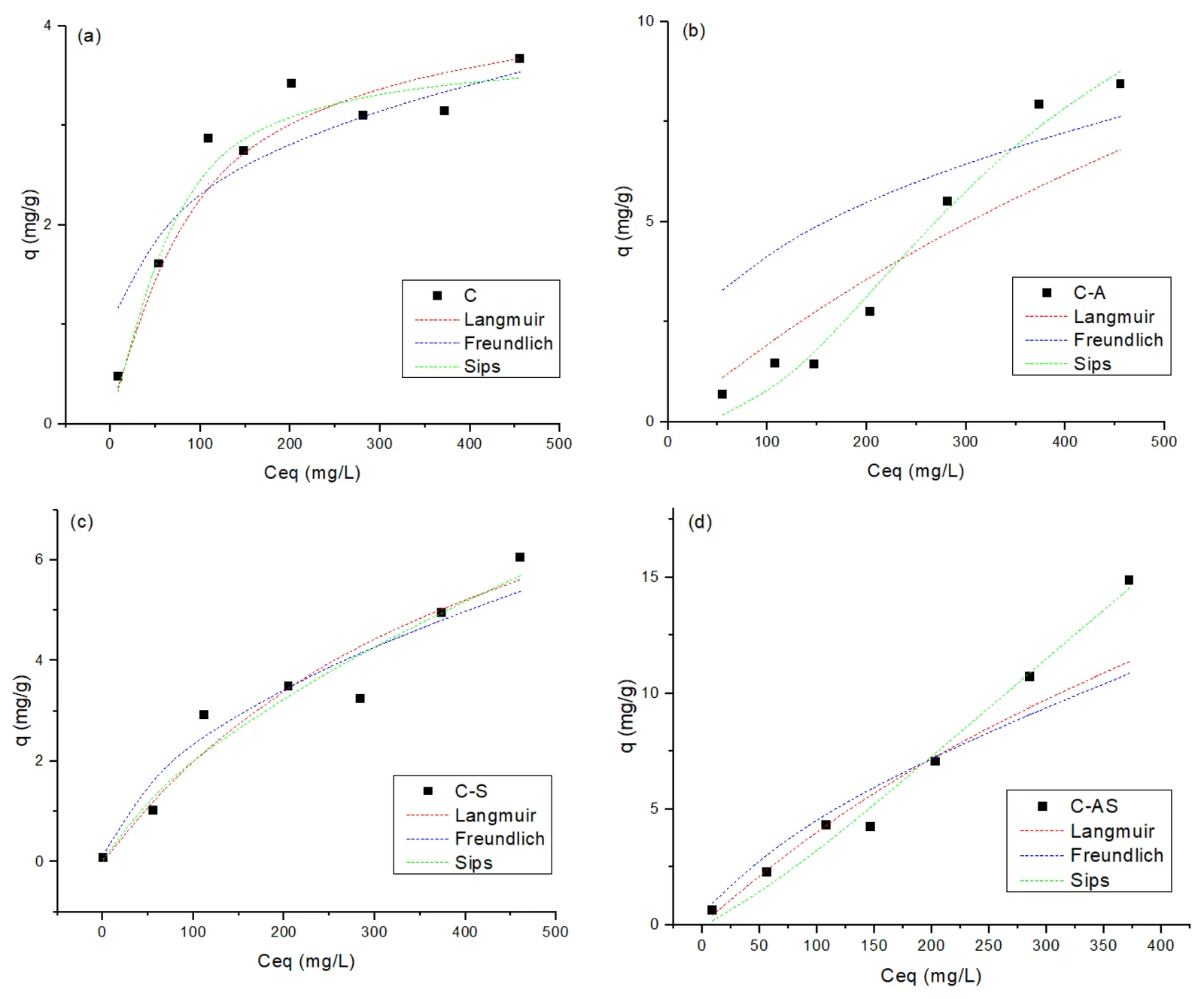
Nonlinear fits of the Langmuir, Freundlich and Sips isotherm models to naproxen equilibrium adsorption data for (**a**) natural clinoptilolite (C), (**b**) acid-activated clinoptilolite (C-A), (**c**) HDTMA-modified clinoptilolite (C-S), and (**d**) acid-activated and subsequently HDTMA-modified clinoptilolite (C-AS). Black squares represent experimental data; red, blue, and green lines indicate Langmuir, Freundlich, and Sips fits, respectively (pH = 6, 25 °C, contact time = 24 h, adsorbent mass = 20 mg). HDTMA: hexadecyltrimethylammonium bromide.

**Figure 13:**
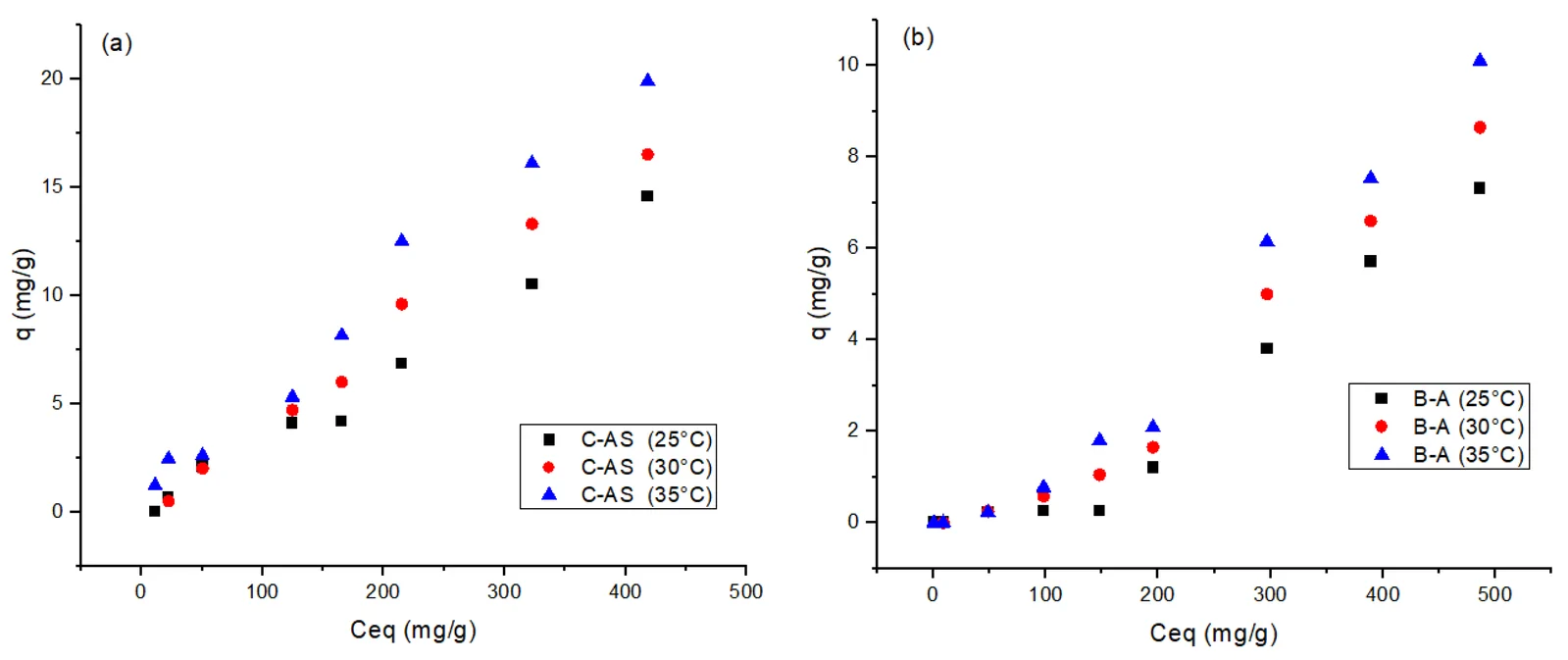
Effect of temperature on naproxen equilibrium adsorption isotherms for (**a**) acid-activated and subsequently HDTMA-modified clinoptilolite (C-AS) and (**b**) acid-activated bentonite (B-A). Black squares and red circles represent experimental data at 25, 30, and 35 °C, respectively (pH = 6, contact time = 24 h, adsorbent mass = 20 mg). HDTMA: hexadecyltrimethylammonium bromide.

**Figure 14:**
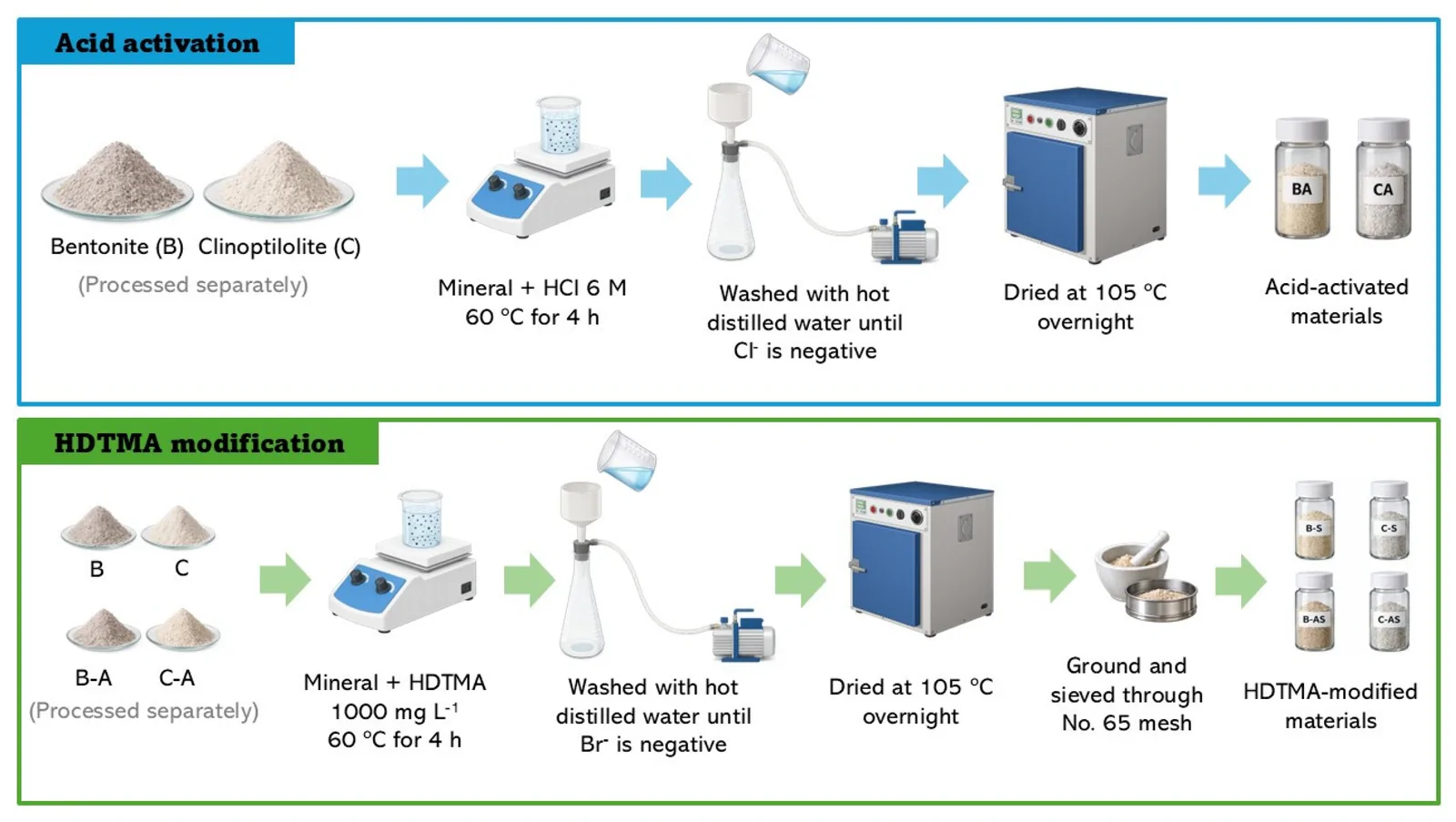
Schematic illustration of the preparation of bentonite- and clinoptilolite-based adsorbents. **Upper** panel: HCl activation of natural bentonite (B) and clinoptilolite (C), followed by washing and drying to obtain BA and CA. **Lower** panel: modification of the natural and acid-activated minerals with hexadecyltrimethylammonium bromide (HDTMA), followed by washing, drying, grinding, and sieving to obtain B-S, C-S, B-AS, and C-AS.

**Table 1:** Textural properties (specific surface area, micropore area, pore volume, and average pore diameter) of clinoptilolite and bentonite before and after sequential HCl and HDTMA treatments.

Material	S_BET_ (m^2^ g^−1^)	S_micro_ (m^2^ g^−1^)	Vp (cm^3^/g)	Dp (nm)
C	22.93	5.22	0.0387	3.09
C-A	82.02	62.19	0.0735	2.15
C-S	27.57	ND	0.0516	4.62
C-AS	23.42	2.39	0.0398	3.91
B	39.18	7.16	0.0985	6.23
B-A	54.89	10.20	0.1304	7.17
B-S	119.25	20.25	0.3315	6.77
B-AS	61.16	1.62	0.1364	5.28

**Table 2:** Kinetic parameters and coefficients of determination (R^2^) obtained from nonlinear fitting of the pseudo-first-order (PFO) and pseudo-second-order (PSO) models to naproxen adsorption data for bentonite- and clinoptilolite-based adsorbents (pH = 6, T = 25 °C, adsorbent mass = 20 mg).

Material	q_PFO_ (mg g^−1^)	k_1_ (min^−1^)	R^2^ (PFO)	χ^2^ (PFO)	q_PSO_ (mg g^−1^)	k_2_ (g mg^−1^ min^−1^)	R^2^ (PSO)	χ^2^ (PSO)
B-A	7.38	0.16	0.8854	0.904	7.21	0.0001	0.9422	0.740
B-S	2.30	0.045	0.8879	1.175	2.49	0.0022	0.9528	0.337
B-AS	2.68	0.059	0.8885	2.923	2.92	0.0031	0.9091	1.313
C	7.15	0.0378	0.9574	0.095	2.64	0.0230	0.7684	0.126
C-A	6.12	0.1299	0.4595	0.767	6.18	0.0106	0.4394	0.392
C-AS	14.78	0.1273	0.6075	1.059	14.97	0.0023	0.7883	0.643
C-S	6.90	0.0637	0.5558	1.303	5.85	0.0247	0.2749	0.433

**Table 3:** Elovich and Avrami fractional-order kinetic parameters for naproxen adsorption onto bentonite and clinoptilolite-based adsorbents.

Material	α (mg g^−1^ min^−1^)	β (g mg^−1^)	R^2^ (Elovich)	χ^2^ (Elovich)	q_e_ (mg g^−1^)	k_AV_ (min^−1^)	n_AV_	R^2^ (Avrami)	χ^2^ (Avrami)
B-A	4093.95	2.281	0.969	0.203	9.93	0.0060	0.129	0.972	0.184
B-S	0.648	2.492	0.996	0.014	2.42	0.0371	0.526	0.999	0.003
B-AS	3.713	1.875	0.984	0.097	5.48	0.0107	0.300	0.989	0.055
C	0.474	1.568	0.947	0.316	2.97	0.0506	0.991	0.985	0.094
C-A	50.13	1.508	0.981	0.119	10.03	0.0055	0.191	0.982	0.110
C-S	56.78	1.372	0.974	0.193	11.07	0.0055	0.192	0.977	0.171
C-AS	49.71	0.518	0.994	0.095	20.29	0.0338	0.267	0.993	0.101

**Table 4:** Isotherm parameters and coefficients of determination (R^2^) obtained from nonlinear fitting of the Langmuir, Freundlich, and Sips models to naproxen equilibrium adsorption data for bentonite- and clinoptilolite-based adsorbents (pH = 6, contact time = 24 h, adsorbent mass = 20 mg).

Material	Q_max_ (mg g^−1^)	K_L_ (L mg^−1^)	R^2^ (Lang.)	χ^2^ (Lang)	K_F_ (mg g^−1^) (L mg^−1^)^n^	n_F_	R^2^ (Fr.)	χ^2^ (Fr.)	q_max_ (mg/g)	Ks ((L/mg)^n^)	n_s_	R^2^ (Sips)	χ^2^ (Sips)
B-A	5.86	0.0011	0.5362	4.387	2.35 × 10^−4^	0.613	0.9329	1.844	8.97	0.003051	3.641	0.9971	6.62
B-S	2.82	0.002	0.6975	0.309	4.88 × 10^−4^	0.672	0.8872	0.339	2.46	0.00396	2.106	0.949	0.273
B-AS	2.45	0.0076	0.9490	0.255	0.0604	1.712	0.9575	0.196	2.07	0.009492	3.782	0.9643	0.165
C	3.95	0.0148	0.9942	0.233	0.1598	1.928	0.9634	0.714	3.73	0.01812	1.229	0.9483	0.214
C-A	11.19	0.0025	0.8267	2.382	0.0061	0.841	0.9739	8.042	12.14	0.00323	2.445	0.9804	2.036
C-S	7.06	0.0056	0.9621	0.964	0.0480	1.525	0.9859	0.673	12.10	0.00180	0.697	0.9378	0.568
C-AS	18.48	0.0031	0.9576	1.739	0.1425	1.208	0.9892	2.625	29.74	0.00253	1.247	0.9846	1.987

**Table 5:** Comparison of naproxen adsorption performance and operating conditions between the present materials and previously reported adsorbents.

Adsorbent	Modification	q (mg g^−1^)	Conditions	Kinetic Model	Isotherm Model	Ref.
Halloysite nanotubes	PAMAM dendrimer-grafted	5.96	pH 6	PSO	Langmuir	[51]
Bentonite	Acid-activated (HCl)	7.3	pH 6, 25 °C	PSO/Avrami	Freundlich	This study
Fe_3_O_4_-supported UiO-66 (Zr-MOF)	Magnetite-modified MOF	14.15	pH 3, 35 °C	PSO	Langmuir	[52]
Clinoptilolite	Sequential acid + HDTMA	14.8/19	pH 6, 25 °C/35 °C	PSO/Avrami	Freundlich	This study
Multi-wall carbon nanotubes	Magnetite-modified	20.75	pH 2, 25 °C	PFO/PSO Bangham	Langmuir	[53]
Activated carbon	ZnCl_2_-activated	106.4	pH 5.5–6, 25 °C	PFO/PSO	Langmuir	[54]

**Table 6:** Standard thermodynamic parameters for naproxen adsorption onto acid-activated and subsequently HDTMA-modified clinoptilolite (C-AS) and acid-activated bentonite (B-A): Gibbs free energy change (ΔG°) at 25, 30, and 35 °C, enthalpy change (ΔH°), and entropy change (ΔS°). HDTMA: hexadecyltrimethylammonium bromide.

Material	ΔG°_25°C_ (kJ mol^−1^)	ΔG°_30°C_ (kJ mol^−1^)	ΔG°_35°C_ (kJ mol^−1^)	ΔH° (kJ mol^−1^)	ΔS° (J mol^−1^ K^−1^)
C-AS	−4.95	−5.63	−6.30	35.28	134.93
B-A	−2.38	−2.96	−3.54	32.25	116.14

## Data Availability

The data presented in this study are available on request from the corresponding authors.
